# Coenzyme A-Dependent Tricarboxylic Acid Cycle Enzymes Are Decreased in Alzheimer’s Disease Consistent With Cerebral Pantothenate Deficiency

**DOI:** 10.3389/fnagi.2022.893159

**Published:** 2022-06-10

**Authors:** Crystal Sang, Sasha A. Philbert, Danielle Hartland, Richard. D Unwin, Andrew W. Dowsey, Jingshu Xu, Garth J. S. Cooper

**Affiliations:** ^1^School of Biological Sciences, Faculty of Science, University of Auckland, Auckland, New Zealand; ^2^Centre for Advanced Discovery & Experimental Therapeutics, Division of Cardiovascular Sciences, School of Medical Sciences, Faculty of Biology, Medicine and Health, The University of Manchester, Manchester Academic Health Science Centre, Manchester, United Kingdom; ^3^Stoller Biomarker Discovery Centre & Division of Cancer Sciences, School of Medical Sciences, Faculty of Biology, Medicine and Health, The University of Manchester, Manchester, United Kingdom; ^4^Department of Population Health Sciences and Bristol Veterinary School, Faculty of Health Sciences, University of Bristol, Bristol, United Kingdom

**Keywords:** tricarboxylic acid cycle (TCA cycle), coenzyme A (CoA), pantothenic acid/vitamin B5, sporadic Alzheimer’s disease, pyruvate dehydrogenase complex, human brain

## Abstract

Sporadic Alzheimer’s disease (sAD) is the commonest cause of age-related neurodegeneration and dementia globally, and a leading cause of premature disability and death. To date, the quest for a disease-modifying therapy for sAD has failed, probably reflecting our incomplete understanding of aetiology and pathogenesis. Drugs that target aggregated Aβ/tau are ineffective, and metabolic defects are now considered to play substantive roles in sAD pathobiology. We tested the hypothesis that the recently identified, pervasive cerebral deficiency of pantothenate (vitamin B5) in sAD, might undermine brain energy metabolism by impairing levels of tricarboxylic acid (TCA)-cycle enzymes and enzyme complexes, some of which require the pantothenate-derived cofactor, coenzyme A (CoA) for their normal functioning. We applied proteomics to measure levels of the multi-subunit TCA-cycle enzymes and their cytoplasmic homologues. We analysed six functionally distinct brain regions from nine sAD cases and nine controls, measuring 33 cerebral proteins that comprise the nine enzymes of the mitochondrial-TCA cycle. Remarkably, we found widespread perturbations affecting only two multi-subunit enzymes and two enzyme complexes, whose function is modulated, directly or indirectly by CoA: pyruvate dehydrogenase complex, isocitrate dehydrogenase, 2-oxoglutarate dehydrogenase complex, and succinyl-CoA synthetase. The sAD cases we studied here displayed widespread deficiency of pantothenate, the obligatory precursor of CoA. Therefore, deficient cerebral pantothenate can damage brain-energy metabolism in sAD, at least in part through impairing levels of these four mitochondrial-TCA-cycle enzymes.

## Introduction

Alzheimer’s disease (AD) is the most common cause of age-related dementia. It is the leading cause of death in the UK (Office for National Statistics, UK Government, 2022) and is only expected to increase in prevalence, given the growth in ageing populations globally. Sporadic AD (sAD) accounts for over 95% of all AD cases worldwide, while all mutations of familial AD are said to account for less than 5% combined (Hampel and Lista, 2012). While the underlying cause(s) of sAD remain unknown, prior publications have established the role of mitochondrial dysfunction in the pathogenesis of neurodegenerative disorders, such as sAD (Cadonic et al., 2016), due to the high energy demand of the brain, which uses ~20% of all energy produced in the body. AD is characterised by the build-up of cerebral plaques, formed from the aggregation of the amyloid β (Aβ) protein and neurofibrillary tangles, composed of the hyperphosphorylated microtubule-associated protein tau (Bloom, 2014).

Many prior publications consider mitochondrial dysfunction in AD to be secondary to plaque and tangle accumulation (Eckert et al., 2011; Manczak and Reddy, 2012; Mondragon-Rodriguez et al., 2013). One related theory is that Aβ binds to the Aβ-binding alcohol dehydrogenase (ABAD) in the mitochondria, leading to mitochondrial dysfunction. Many believe that the binding of Aβ to ABAD prevents ABAD from binding to NAD^+^, thus reducing the mitochondrial membrane’s permeability for electron carriers, and lowering mitochondrial enzyme activity (Yan and Stern, 2005). Additional hypotheses have been generated following the discovery of amyloid oligomers (Goldsbury et al., 1999) and that they can exert toxicity towards neurons *via* several molecular pathways including interactions with α-synuclein (Tsigelny et al., 2008) but the exact mechanism is still unknown. Tau pathology is similarly believed to contribute to mitochondrial dysfunction, albeit through damaging axonal transport which leads to abnormal mitochondria distribution (Kopeikina et al., 2011; Shahpasand et al., 2012). However, mitochondrial dysfunction has been witnessed in triple transgenic mice (Yao et al., 2009) and *C. elegans* (Teo et al., 2019) at even relatively low Aβ levels, prior to plaque formation, which raises the question of whether the protein aggregation may be a consequence of mitochondrial dysfunction, and whether there is a primary role of metabolic defects in AD. Decreased energy metabolism has been shown to lead to the build-up of Aβ (Gabuzda et al., 1994) and the hyperphosphorylation of tau (Planel et al., 2004).

Several studies have shown significant global reductions in the level of pantothenic acid in the brains of multiple forms of neurodegeneration, including Huntington’s disease, Parkinson’s disease, and AD, which may be causative of the mitochondrial dysfunction (Patassini et al., 2019; Xu et al., 2020). Data demonstrating pantothenic acid reduction measured in nine AD cases and age-matched controls (from Xu et al., 2020) are presented in Table 1. Defective dietary intake is an unlikely factor in the decreased level of pantothenic acid in sAD brains, as the average adult only requires 5 mg daily which can easily be acquired from the typical Western diet (NIH, 1998). These observations indicate that the cause for the decreased pantothenic acid could possibly be a defect in cerebral pantothenic acid transporters such as the SLC5A6 (Patassini et al., 2019).

**Table 1:** Level of pantothenate in *post-mortem* human brain in Alzheimer’s disease and age-matched controls.

**Brain region**	**Control mean (μmol/kg tissue)**	**Case mean (μmol/kg tissue)**	**Fold change (AD vs. control)**	***p*-value**
HP	33.9 (20.8–47.0)	15.4 (10.2–20.5)	0.5	0.0079
ENT	38.2 (26.8–49.5)	15.2 (10.3–20.0)	0.4	0.0006
MTG	36.5 (25.3–47.7)	18.2 (12.8–23.6)	0.5	0.0037
CG	33.7 (23.3–44.2)	15.3 (8.6–21.9)	0.5	0.0034
SCx	43.5 (29.9–57.0)	17.1 (9.8–24.3)	0.4	0.0011
MCx	42.5 (28.5–56.5)	14.6 (8.3–20.8)	0.3	0.0007
CB	55.4 (34.0–76.8)	25.4 (19.3–31.4)	0.5	0.0067
Overall	40.5 (35.8–45.2)	17.3 (15.2–19.2)	0.4	3E-15

*Mean (±95% CI) values of pantothenate concentration (μmol/kg wet-tissue) for each brain region in controls and AD cases, where statistical significance was calculated using multiple two-tailed *t*-tests. Abbreviations: HP, hippocampus; ENT, entorhinal cortex; MTG, middle temporal gyrus; CG, cingulate gyrus; SCx, sensory cortex; MCx, motor cortex; CB, cerebellum*.

Pantothenic acid is the precursor for coenzyme A (CoA; Hayflick, 2014), which is added to acetyl-CoA by the pyruvate dehydrogenase complex (PDHC; Patel and Korotchkina, 2006). Acetyl-CoA is an obligatory cofactor for ~4% of all mammalian enzymes, and participates in the TCA cycle, fatty acid synthesis, and β-oxidation (lipid catabolism) among other energy-producing pathways (Daugherty et al., 2002). Acetyl-CoA is also the acetyl donor for the formation of the major neurotransmitter, acetylcholine (Venco et al., 2014), and plays a central role in processes such as biosynthesis of haeme and myelin (Xu et al., 2020). Haeme biosynthesis begins with the condensation of glycine and succinyl-CoA in the mitochondria, and elevated levels of free haeme and haemoglobin have been found in AD brains (Philbert et al., 2021).

Defects in the metabolic pathway for the synthesis of CoA from pantothenic acid have been postulated to cause neurodegeneration (Houlden et al., 2003); notably, a mutation in pantothenate kinase 2 (PANK2) causes neuronal cell death in both the globus pallidus and substantia nigra while eliciting iron accumulation in both these regions (Hayflick, 2014). Pantothenate kinase-associated neurodegeneration (PKAN) exhibits some of the symptoms also seen in dementia but without the pathological hallmarks of AD, namely the accumulation of plaques and tangles in the brain. Thus, in the case of PKAN, Aβ/tau aggregation is evidently not required for neurodegeneration, emphasising the pathogenic role of the pantothenic acid deficiency itself. Clinical trials treating PKAN with 4’-phosphopantetheine and other pantothenic acid supplements have been shown to be successful, reflecting CoA deficiency itself as a potential culprit in the pathogenesis of PKAN and also, potentially, in AD (Jeong et al., 2019). The oxidative stress observed in AD brains has been observed in model organisms with a pantothenic acid deficiency (Qian et al., 2015; Wang et al., 2016), further consistent with a role of this deficiency in AD pathogenesis.

Here, we aimed to test the role of cerebral pantothenic acid deficiency in the pathogenesis of AD by measuring the levels of enzymes and enzyme complexes implicated in the TCA cycle, to assess the potential roles of altered energy-producing pathways in the pathogenesis of AD. We used liquid chromatography-mass spectrometry (LC-MS) to measure protein levels (Xu et al., 2019) in six anatomically and functionally distinct regions of the *post-mortem* human brain, including three which undergo severe neuronal change in sAD [hippocampus (HP), entorhinal cortex (ENT), and cingulate gyrus (CG)]; two which are moderately affected [sensory cortex (SCx) and motor cortex (MCx)]; as well as the cerebellum (CB) which was considered to be largely spared but has now been shown also to be affected (Braak and Braak, 1991). Eighteen human brains with short *post-mortem* delays were analysed, including nine cases of sAD and nine matched controls. We applied mixed hypothesis-generating and pathway-targeted methodologies with Bayesian modelling to quantitate case-control differences in the levels of various proteins in the brain.

## Methods

The methods employed in this study of *post-mortem* brains affected by AD and controls were as previously described (Schonberger et al., 2001; Xu et al., 2019; Philbert et al., 2021), with steps unique to the current study summarised in this section. The methods are described in greater detail, in referenced prior publications (Xu et al., 2019; Philbert et al., 2021) and are presented here in the Supplementary Information.

### Ethics and Acquisition of Human Brains

Experiments were performed at the Universities of Auckland and Manchester, following relevant ethics regulations and guidelines. This study of the *post-mortem* brain was approved by the University of Auckland Human Participants Ethics Committee with informed consent from all families, as previously described (Schonberger et al., 2001; Xu et al., 2019; Philbert et al., 2021).

### Brain Dissection, Sample Acquisition, and Histopathological Diagnosis

Brains were dissected as described (Supplementary Methods). Cases had *ante-mortem* clinical evidence of dementia whereas age, sex, and *post-mortem* delay (PMD)-matched controls did not. For this proteomic analysis, aliquots of grey matter were dissected from six regions, HP, ENT, CG, SCx, MCx, and CB.

Aliquots of 100 ± 5 mg wet weight were dissected from each region, stored at −80°C until analysis, and otherwise treated as described.

A consultant neuropathologist diagnosed or excluded AD by applying the Consortium to Establish a Registry for Alzheimer’s Disease (CERAD) criteria and determined the neuropathological severity by assigning the Braak stage, and amyloid load by applying the 2013 consensus National Institute on Aging Alzheimer’s Association guidelines. Grouped case data are shown in Table 2, and individual case data in Supplementary Table 1. One control patient had neuropathological findings consistent with AD (Braak Stage II) and was therefore diagnosed with the pre-clinical disease but retained in the analysis: this finding is consistent with the known frequency of pre-symptomatic AD in similarly aged groups in the study population.

**Table 2:** Case-control study of *post-mortem* human brain in Alzheimer’s disease: group characteristics.

**Variable**	**Control**	**Alzheimer’s disease**
Number	9	9
Age	70.1 (6.7)	70.3 (7.1)
Male sex, *n* (%)	5 (55.6)	5 (55.6)
PMD (h)	9 (5.5–13.0)	7 (4.0–12.0)*
Brain-weight (g)	1,260 (1,094–1,461)	1,062 (831–1,355)*
Plaques, *n* (%)	1 (11)	9 (100)**
Tangles, *n* (%)	1 (11)	9 (100)**

Values are age, mean (SD); PMD and brain-weights, median (range): *P < 0.05, **P < 0.0001 compared with corresponding Control value; all other differences are non-significant. Abbreviation: PMD, *post-mortem* delay.

### Protein Extraction and Preparation for iTRAQ Labelling

Protein extraction and preparation for iTRAQ protein quantification was performed as previously described with each brain region analysed independently. A detailed description of the methodology is presented in the Supplementary Methods.

### HpHRP Fractionation

HpHRP was performed as previously described for the LC-MS analysis and as detailed in the Supplementary Methods.

### Low-pH LC-MS Data Acquisition

Data acquisition by LC-MS was performed as previously described and detailed in the Supplementary Methods.

### Data Processing

The data-processing methodologies applied in this study were as previously described and detailed in the Supplementary Methods. Proteins relevant to the underlying experimental hypothesis were identified by the authors’ prior field-related knowledge of relevant protein pathways as listed in the “Introduction” Section and were assessed by Bayesian probability distribution plots for each protein, along with probability distribution plots for each case.

### Data Availability

All fully processed data used in this case-control study are available *via* the Supplementary Data associated with our previous articles and online in a searchable format, along with probability distribution plots for each protein at: www.manchester.ac.uk/dementia-proteomes-project.

## Results

We report measurements of 33 proteins that are components of TCA-cycle enzymes in cerebral tissues from six functionally distinct brain regions, in nine cases with sAD and nine matched controls. Study-group characteristics are summarised in Table 2, with additional individual patient data in Supplementary Table 1.

**Figure 1:**
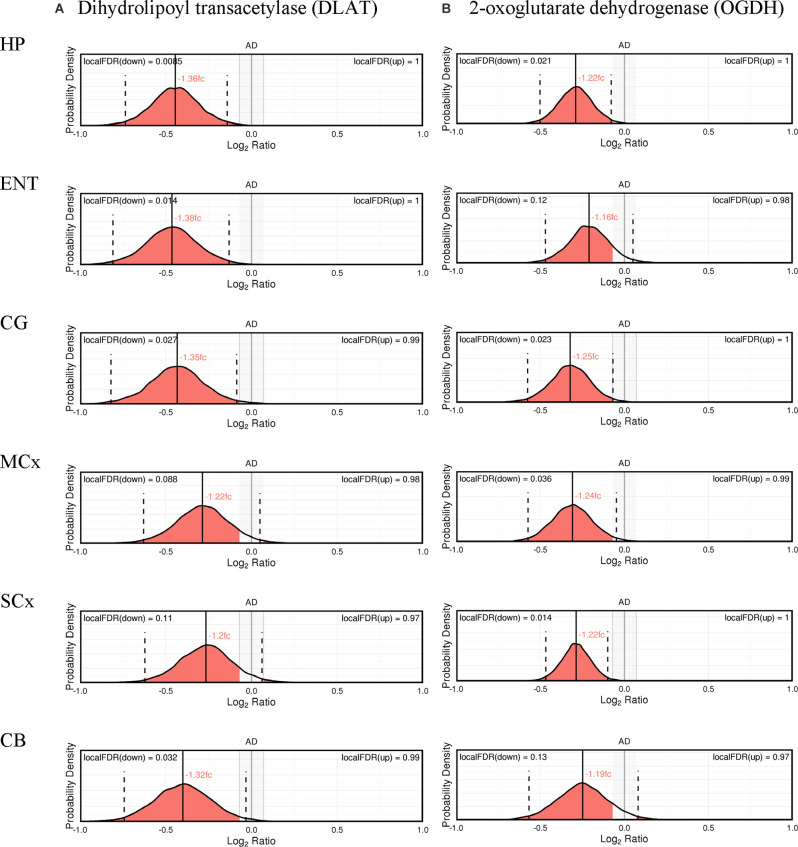
Expression of cerebral dihydrolipoyl transacetylase **(A)** and 2-oxoglutarate dehydrogenase **(B)**.

**Figure 2:**
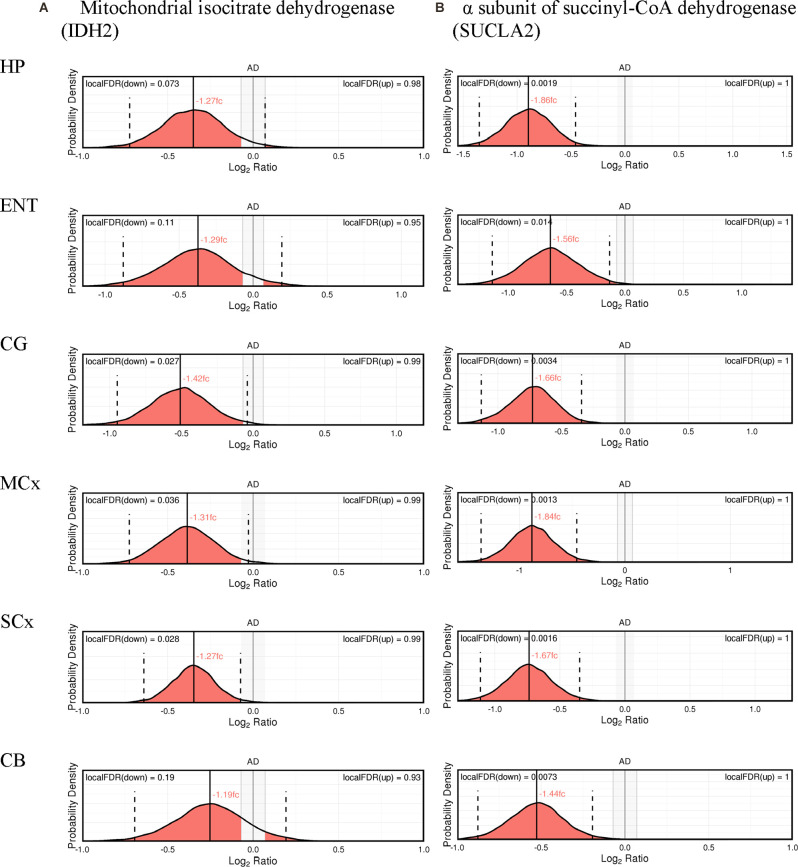
Expression of cerebral mitochondrial isocitrate dehydrogenase **(A)**, and the α subunit of succinyl-CoA dehydrogenase **(B)**.

The expression probability distributions of these proteins in six brain regions are shown in Figures 1, 2, with the corresponding Bayesian differential quantification data as shown in Table 3. The different components of an exemplary expression probability distribution diagram are shown in Supplementary Figure 1 for guidance. Each plot shows the probability distribution along with the most likely mean-expression ratio, and the calculated local false-discovery rate (FDR) for each protein molecule that had a differential expression between cases and controls of at least 5%. Shown in Supplementary Figures 2, 3 are the Bayesian probability distributions for the estimated levels of DLAT and OGDH in each individual sample in this study, with respect to the mean-control level.

**Table 3:** Multiregional Bayesian-differential quantification for cerebral dihydrolipoyl transacetylase, 2-oxoglutarate dehydrogenase, mitochondrial isocitrate dehydrogenase and the α subunit of the succinyl-CoA synthetase expression.

**DLAT Brain region**	**Peptides**	**Spectra**	**Log2 (fc)**	**Lower**	**Upper**	**Local FDR**
Hippocampus	15	89	−0.444	−0.735	−0.142	0.0085
Entorhinal cortex	18	86	−0.491	−0.836	−0.136	0.0128
Cingulate gyrus	15	76	−0.478	−0.881	−0.053	0.0278
Motor cortex	16	68	−0.297	−0.666	0.032	0.0909
Sensory cortex	17	110	−0.247	−0.573	0.079	0.1178
Cerebellum	17	78	−0.380	−0.711	−0.0455	0.0311
**OGDH Brain region**						
Hippocampus	16	67	−0.291	−0.503	−0.077	0.0212
Entorhinal cortex	20	57	−0.261	−0.531	0.013	0.0742
Cingulate gyrus	10	44	−0.289	−0.510	−0.059	0.0277
Motor cortex	16	45	−0.324	−0.575	−0.076	0.0243
Sensory cortex	20	72	−0.284	−0.473	−0.099	0.0148
Cerebellum	16	59	−0.261	−0.573	0.059	0.1008
**IDH2 Brain region**						
Hippocampus	20	118	−0.350	−0.722	0.070	0.0734
Entorhinal cortex	26	155	−0.392	−0.944	0.153	0.1038
Cingulate gyrus	25	143	−0.428	−0.814	−0.048	0.032
Motor cortex	24	81	−0.370	−0.712	−0.006	0.0441
Sensory cortex	19	136	−0.343	−0.619	−0.077	0.0265
Cerebellum	20	94	−0.223	−0.631	0.205	0.2156
**SUCLA2 Brain region**						
Hippocampus	18	69	−0.896	−1.351	−0.458	0.0019
Entorhinal cortex	18	64	−0.714	−1.266	−0.206	0.0108
Cingulate gyrus	18	58	−0.580	−0.969	−0.216	0.0061
Motor cortex	16	54	−0.668	−1.144	−0.199	0.0085
Sensory cortex	19	76	−0.723	−1.088	−0.351	0.001
Cerebellum	24	86	−0.568	−1.015	−0.091	0.0195

**Figure 3:**
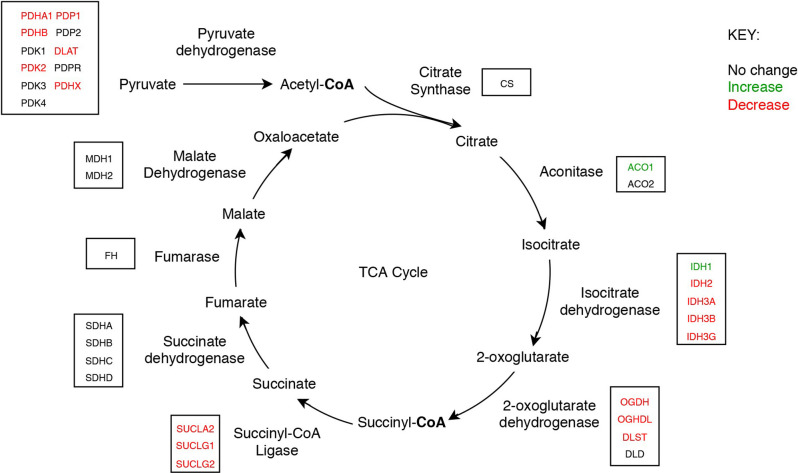
The tricarboxylic acid cycle showing concentrations of mitochondrial enzyme molecules whose levels were found to be altered in sAD brain in this study.

Nine protein components of the pyruvate dehydrogenase complex (PDHC) were measured (Figure 3). This enzyme catalyses the reaction directly before the 2-carbon moieties enter the TCA cycle, the condensation of acetyl-CoA and oxaloacetate to form citrate. Significant probability of reductions was found in all three subunits of the PDHC through decreased concentrations of six proteins (PDHA1, PDHB, EC 1.2.4.1; PDK2, EC: 2.7.11.2; PDP1, EC 3.1.3.43; DLAT, EC 2.3.1.12; PDHX). E2 (DLAT) showed the greatest perturbation, with decreases in protein levels in five of the six brain regions examined showing on average a 1.3-fold decrease and the smallest local FDR of 0.85% in the HP: the only exception was the SCx where no significant probability of change was observed.

Up to 1.2- and 1.27-fold reductions were also measured in both the α and β subunits of E1 (PDHA1, PDHB) respectively and reductions in certain regulatory enzymes of E1, PDK2, and PDP1, as well as in PDHX, the protein which links E3 to E2 (Zhou et al., 2001), indicating that there was also a decrease in the protein levels of E3. No significant probability of changes was present in PDPR, PDK1 and PDK3. PDK1, and PDK3 are both paralogs of PDK2, which is ubiquitously expressed (Bowker-Kinley et al., 1998), while PDPR is the regulatory unit of PDP. All three enzymes are closely associated with enzymes that show a significant probability of decreases with local FDR ranging from 2% to 10%.

We found that a similar decrease in protein levels was present in the 2-oxoglutarate dehydrogenase (α-ketoglutarate dehydrogenase) complex (OGDHC), which is consistent with expectation given that it is structurally related to the PDHC. Four proteins of the OGDHC were measured, including the E1 subunit OGDH (EC 1.2.4.2), the E1-like subunit OGDHL, the E2 subunit DLST (EC 2.3.1.61), and E3 DLD (EC 1.8.1.4; Figure 3). A significant probability of reduction was present in five of the six brain regions for both OGDH and OGDHL, with the smallest local FDR of 1.5% for the OGDH in the SCx and 0.15% for the OGDHL in the HP while no significant probability of changes seen in the CB for E1 of OGDH and in the MCx for OGDGL. No significant probability of changes was seen in the concentration of DLD, but the DLST showed a decreased concentration in the ENT with a local FDR of 8%.

Of the two isozymes of aconitase (EC 2.3.3.1), the cytoplasmic isozyme encoded by ACO1 was increased in concentration in the HP and ENT with a local FDR of 8% for both while no change was seen in the mitochondrial isozyme encoded by ACO2.

In isocitrate dehydrogenase (IDH, EC 1.1.1.41), catalytic, structural, and regulatory subunits alike were decreased in AD brains, compared with controls in various regions. All five proteins of IDH showed a significant probability of perturbations in concentration (Figure 3). All three subunits of IDH, which use NAD^+^ as a cofactor (IDH3A, IDH3B, and IDH3G) were decreased in concentration. Most notably, IDH3G, the regulatory subunit of IDH, was decreased in all six regions of the brain with the smallest FDR in the HP of 1%, clearly indicating the decrease of IDH in the brain of AD cases and perturbed energetic pathways. Of the two IDH isoforms which use NADP^+^ as a cofactor, the mitochondrial IDH2 was decreased in concentration in four regions while the cytoplasmic IDH1 was increased in concentration in the CG with a local FDR of 1.7%.

All three subunits of succinyl-CoA synthetase (succinyl-CoA ligase), SUCLA2 (EC 6.2.1.5), SUCLG1, and SUCLG2 (EC 6.2.1.4) were decreased in concentration in AD cases (Figure 3). SUCLA2 encodes the α subunit of succinyl-CoA synthetase while SUCLG1 and SUCLG2 encode the α and β subunits of the succinyl-CoA GDP/ADP-forming ligase respectively. This enzyme catalyses the reversible conversion of succinyl-CoA to succinate as well as substrate phosphorylation. Of the heterodimeric succinyl-CoA synthetase, the α subunit is invariant while the β subunits are substrate-specific. Notably, both SUCLA2 and SUCLG1 had a significant probability of a decrease in concentration in all six brain regions examined with local FDR values ranging from 0.1% to 2%. This perturbation may be indicative also of perturbations in fatty acid metabolism in AD cases, as metabolised fatty acids enter the TCA cycle as succinyl-CoA *via* these enzymes.

No enzymes following succinyl-CoA synthetase in the TCA cycle showed perturbations in concentration.

## Discussion

The results have clearly shown that there exists a significant probability of perturbations in the protein concentrations of several TCA cycle enzymes and enzyme complexes in AD brains, notably a 1.84-fold decrease in the concentration of SUCLA2 in the MCx. Enzymes and enzyme complexes in the first half of the TCA cycle are most significantly affected. It is evident that, overall, there is a decrease in the concentration of TCA-cycle components and thus of the energy-producing pathway itself. These perturbations may well be closely related to the pathogenesis of AD to impact brain function, given the high energy requirement of the brain.

The TCA cycle has a key role in aerobic respiration, and the decreased protein concentrations will lead to decreased metabolites in each step and result in less production of electron carriers for the oxidative phosphorylation pathway. The PDHC catalyses the conversion of pyruvate to acetyl-CoA, thus linking the glycolytic pathway to the TCA cycle in the breakdown of carbohydrates (Zhou et al., 2001; Patel and Korotchkina, 2006). IDH and OGDH catalyse the rate-limiting steps in the TCA cycle and thus act as loci for regulation. IDH catalyses the conversion of isocitrate to 2-oxoglutarate and OGDH converts 2-oxoglutarate to succinyl-CoA. IDH is allosterically regulated by ATP and NADH while OGDH is allosterically regulated by ATP, NADH, and its product succinyl-CoA. Succinyl-CoA synthetase catalyses the reversible reaction between succinyl-CoA and succinate.

While the decrease in the concentration of enzymes is not a direct indication of decreased enzyme activity, the latter has also been observed in TCA cycle enzyme complexes (Bubber et al., 2005). The evidence of both decreased enzyme concentrations and activity is sufficient to demonstrate the existence of decreased outputs of the TCA cycle. A reduction in the level of PDHC results in fewer carbon moieties entering the TCA cycle due to decreased acetyl-CoA production. This will go on to result in decreased flux for the following reactions. The decrease in concentrations and activity of the three enzymes which follow is likely a result of this decreased flux which will result in decreased ATP production overall.

The global reduction in pantothenic acid, which is required for the synthesis of the cofactor CoA, is likely linked to the decreased level of certain proteins in the TCA cycle enzymes. Previous studies have reported reduced activities in the PDHC in various regions of AD brains, accompanied by a reduction in the activity of choline acetyltransferase (Perry et al., 1980; Sorbi et al., 1983; Sheu et al., 1985). Whether the reduced output of acetyl-CoA or acetylcholine is secondary to the other is in contention, but the correlation between the two has been clearly established (Perry et al., 1980).

To our knowledge, no prior publications have associated both the decreased levels of PDHC proteins and ChAT with the global pantothenic acid deficiency in sAD brains. Many prior hypotheses for the reduction in TCA cycle enzymes included impaired processing of the precursors of the enzymes and the loss of cholinergic neurons enriched in either the PDHC or OGDHC (Perry et al., 1980). The first is unlikely given that proteomic studies have failed to find an increase in the protein levels of the enzyme precursors (Mastrogiacoma et al., 1996b). The second has been observed through decreased levels of ChAT in AD brains but the metabolic perturbations were interpreted to reflect consequence rather than a cause.

We propose that the decreased TCA cycle protein concentrations may be the result of a pantothenic acid deficiency in the brain, which has been observed in patients with AD (Xu et al., 2020). There is substantive evidence for a concentrated store of pantothenic acid in the white matter consistent with the high demand for CoA in myelin biosynthesis (Ismail et al., 2020). The demyelination in multiple dementias (such as Alzheimer’s and Huntington’s diseases; Patassini et al., 2019; Xu et al., 2020) suggests that reduced CoA levels may contribute to the pathogenesis of AD and thus decreased activities of both the PDHC and ChAT. Thus, both the loss of cholinergic neurons and TCA cycle enzymes may well be due to the pantothenic acid deficiency, and the loss of cholinergic neurons could further decrease the protein levels of TCA-cycle enzymes. Brains deficient in TCA-cycle enzymes are observed to increase the production of reactive oxygen species (ROS; Klivenyi et al., 2004), which are known to play a significant role in the pathogenesis of neurodegenerative diseases such as AD (Manoharan et al., 2016). This is one potential mechanism by which pantothenic acid deficiency could result in AD.

In examining the relationship between CoA and each of the proteins which show a decrease in concentration, we find that all enzymes which show a significant probability of a decrease in protein concentrations, use CoA as a co-factor in their catalytic mechanisms. The PDHC directly uses CoA in converting pyruvate to acetyl-CoA where CoA is attached to substrate by E2, which coincidentally shows the largest decrease in the concentration of all PDHC proteins. In the OGDHC, which is structurally similar to the PDHC, the same mechanism occurs where CoA is attached by E2. However, unlike the PDHC, the protein concentration of E1 had the greatest probability of the decrease and this is likely due to the significant probability of decrease in flux from the PDHC. The E2 subunit also demonstrates a decrease, which is likely associated with the CoA deficiency. Pyruvate dehydrogenase deficiency is known to result in neuropathy due to abnormal mitochondrial metabolism (Patel et al., 2012), supporting the view that defective metabolic pathways might well be causative of neurodegeneration in AD, through impairment of cerebral energy metabolism.

Some prior publications have associated the decrease of both the PDHC and OGDHC with a thiamine deficiency (Gibson et al., 1988; Butterworth and Besnard, 1990), but significant thiamine deficiency sufficient to cause neurodegeneration, would almost certainly manifest in both clinical and *post-mortem* examinations with typical signs of Beriberi. Additionally, no evidence of thiamine deficiency was reported in any of the AD cases in this investigation (Xu et al., 2019). Every AD case in this proteomic analysis showed clear evidence of substantively elevated brain Aβ levels in all six brain regions studied, consistent with a diagnosis of AD, along with compelling neuropathological findings sufficient to support the diagnosis of AD in every case. The *ante-mortem* reports in every case also supported the diagnosis of AD but not the diagnosis of thiamine deficiency. Therefore, all cases that we are studying here are bona fide cases of AD, and none of them had signs or symptoms of thiamine deficiency. It is true that some cases of AD may manifest mild indexes of thiamine deficiency as mentioned in Lu’o’ng and Nguyen (2011), but that is likely to be caused in such cases by a secondary, nutritional deficit in patients who were unable to maintain a sufficient dietary intake because of the impacts of AD. Treatment with high-dose thiamine has been shown to have mild benefits in patients with AD (Mastrogiacoma et al., 1996a), but the same study found no change in brain-free thiamine levels, only a decrease in thiamine diphosphate which the authors postulated could be due to the cerebral cortical deficiency of ATP in AD. In contrast, there is substantive evidence for cerebral pantothenic acid deficiency in AD (Xu et al., 2020) as well as in other neurodegenerative diseases such as Huntington’s (Patassini et al., 2019) and Parkinson’s disease (Scholefield et al., 2021). Therefore, changes in the status of PDHC and OGDHC observed in our study are not caused by thiamine deficiency, but likely by pantothenic acid deficiency. Similarly, other publications have associated AD with a cobalamin or folate deficiency (Chen et al., 2015) but these perturbations are not reflected in the AD cases investigated here. Both folate and cobalamin deficiencies would manifest as macrocytic anemia in patients but again this was not observed in the AD cases investigated. The significant difference between cobalamin, folate, and thiamine deficiencies compared to a pantothenate deficiency is that pantothenate is widely abundant in the typical Western diet and patients are unlikely to suffer a pantothenate deficiency due to their diet.

The final enzyme demonstrating decreased protein concentration is succinyl-CoA synthetase, whose reverse reaction in converting succinate to succinyl-CoA, which can then be a substrate for haeme synthesis, also uses CoA as a cofactor. Haeme synthesis begins in the mitochondria with the condensation of succinyl-CoA and glycine. Neuronal haeme biosynthesis is altered in multiple neurodegenerative diseases such as Parkinson’s disease (Freed and Chakrabarti, 2016), and the importance of haeme in normal mitochondrial function has been established (Fukuda et al., 2017).

The observed decrease in succinyl-CoA synthetase may also be attributed to decreased fatty acid synthesis. Given the close association between the TCA cycle and fatty acid metabolism, where fatty acid-derived moieties can enter the TCA cycle either as acetyl-CoA or succinyl-CoA, it is likely that the AD brain would attempt to increase fatty acid oxidisation to satisfy its energy demand, and concurrently decrease fatty acid synthesis to avoid undergoing a futile cycle. This behavior is consistent with the hypothesis of increased alternate energy-producing pathways to meet the energy demand of the brain (Hoyer and Krier, 1986).

Studies have reported various relationships between fatty acids and the two hallmarks of AD, the accumulation of Aβ and tau in the brain. Lipids play a role in the synthesis, misfolding, and polymerisation of Aβ, as well as in the phosphorylation and polymerisation of tau (Han, 2010; Di Paolo and Kim, 2011). Alois Alzheimer also described a little-known third hallmark of AD, namely that brains affected by AD have a higher occurrence of adipose inclusions, consistent with defects in cerebral lipid metabolism (Foley, 2010). Defective lipid metabolism is now confirmed to relate to AD through the discovery that carriers of the apolipoprotein E type 4 (APOE4) allele, involved in lipid transport and metabolism, convey an elevated risk of both sporadic and late-onset familial AD (Corder et al., 1993). We conclude that the perturbations of proteins involved in fatty acid metabolism should be further explored.

While significant probabilities of decreases in the protein concentrations of four TCA-cycle enzymes are evident here, no measurable changes were present in the concentration of citrate synthase or any of the enzymes after succinyl-CoA synthetase in the cycle. This provides further evidence that pantothenic acid deficiency may well be responsible for the metabolic perturbations, as no enzymes following succinyl-CoA synthetase use CoA as a cofactor. While acetyl-CoA is a substrate in the reaction catalysed by citrate synthase in the condensation with oxaloacetate to citrate, no net CoA is used in this reaction, hence providing a potential explanation for why the concentration of citrate synthase measured remained unchanged in AD brains in this study.

The increases in protein concentrations of ACO1 and IDH1 might be attributable to their role in the production of the redox carrier NADPH. This is similarly reflected in our observation of increased concentrations of enzymes in the pentose phosphate pathway (PPP), for which one primary function is the production of NADPH to alleviate oxidative stress. An increase in the activity of 6-phosphogluconate dehydrogenase and glucose-6-phosphate dehydrogenase has also been reported in AD brains (Palmer, 1999). ROS are known to increase in AD brains (Aslan and Ozben, 2004) and the increased production of NADPH is likely a response to this increase in ROS. Glutathione, which is mostly present in the reduced state within the cell, is able to reduce ROS and in the process itself become oxidised to glutathione disulphide, which then requires NADPH to reconvert it to the reduced form (Kosower and Kosower, 1978).

Given that sAD is a late-onset disease, where 95% of cases are diagnosed after the age of 65, it is worthwhile to examine the similarities shared between ageing and AD brains. Using this line of reasoning, two potential AD drug candidates, CMS121 and J147, both of which increase the level of acetyl-CoA, had been identified which alleviates the symptoms of the ageing brain. Both CMS121 and J147 act by inhibiting acetyl-CoA carboxylase I to increase mitochondrial acetyl-CoA. The administration of these two compounds to rapidly ageing Samp8 mice can reduce age-related cognitive dysfunction (Currais et al., 2019). The increased acetyl-CoA resulting from these compounds regulates metabolites in the energy pathways including the TCA cycle. This observation directly supports our idea that decreased acetyl-CoA could contribute to the pathogenesis of AD. This indicates the sharing of metabolic pathways between ageing and AD brains which might be targeted by increasing acetyl-CoA. We postulate that the observed pantothenic acid deficiency could further exacerbate the effects of already decreased acetyl-CoA levels in ageing brains, which could well provide a mechanism for increased risk of age-related increases in AD-evoked neurodegeneration. Decreased glucose metabolism is also observed in both AD and normal ageing brains where the decrease in cerebral glucose uptake is seen to be more severe in AD brains and precedes the symptoms of AD (Cunnane et al., 2011; Caldwell et al., 2015). These observations provide further evidence that AD is a metabolic disease which is directly related to the two hallmarks of AD, Aβ and tau protein accumulations. Glycation of tau enhances the formation of paired helical filaments and glycation of Aβ has been found to enhance its aggregation* in vitro* (Sasaki et al., 1998). Decreased glucose metabolism has also been shown to lead to decreased O-GlcNac glycosylation of tau, and the precursor of Aβ, amyloid precursor protein (APP; Yuzwa and Vocadlo, 2014). There is a proven reciprocal relationship between tau O-GlcNac modification and phosphorylation, where decreased O-GlcNac modifications on tau corresponds to hyperphosphorylation and thus the aggregation of tau (Dias and Hart, 2007). Similarly, with APP it has been shown that decreased O-GlcNac modification results in more production of Aβ 40 and 42 and plaque formation (Kim et al., 2013). These provide further evidence that tau and Aβ should not be considered the cause of AD but rather a symptom of the metabolic defects which are causative of AD.

Decreased acetyl-CoA also decreases histone acetylation in the brain, especially of H3K9, contributing to memory loss (Bradshaw, 2021), another key symptom of AD. It was observed in rats that learning results in a transient increase of histone acetylation in the hippocampus (Levenson et al., 2004), which happens to be one of the most perturbed regions of the brain in AD cases. Acetyl-CoA is the acetyl donor for the histone acetylation process and is thus directly implicated in gene expression. Decreased histone acetylation decreases chromatin accessibility to the basal transcription machinery and results in decreased transcription and translation of proteins. Inhibitors of the histone deacetylase HDAC3 have been shown to be effective in reversing AD pathologies, specifically dephosphorylation of tau and decreased levels of Aβ proteins, in triple transgenic mice (Janczura et al., 2018). Histone deacetylation occurs rapidly when acetyl-CoA synthesis is compromised (Galdieri and Vancura, 2012). This means the likely decrease in acetyl-CoA resulting from pantothenic acid deficiency in AD, may also result in defects in metabolic pathways at the transcriptional level, resulting in decreased protein concentrations.

In addition to regulation *via* histone acetylation, the expression of TCA cycle enzymes and enzyme complexes may directly be regulated by metabolites and enzymes of the cycle. The increased expression of the *PDHA1* gene with increased glucose concentration provided the first evidence for transcriptional level regulation of a TCA cycle (Tan et al., 1998). Since then, there has been increasing evidence to suggest that both metabolic enzymes and metabolic co-factors such as acetyl-CoA play a direct role in regulating the expression of TCA cycle enzyme and enzyme complexes (Shi and Shi, 2004). PDK4, which is highly expressed in heart, liver, and skeletal muscle mitochondria, has been found to be regulated by insulin concentration at the transcriptional level, where the PDK4 gene possesses three insulin response sequences which are binding sites for transcription factors FOXO1 and FOXO3 (Jeong et al., 2012). We propose an equivalent mechanism may exist for the many other enzymes and enzyme complexes of the TCA cycle where there are specific sequences in the promoter that act as binding sites for transcription factors whose expression may be regulated by acetyl-CoA. Evidence has been observed for the transcription factor FadR that regulates the expression of fatty acid metabolism enzymes in *E. coli*. FadR has a binding site for acyl-CoA which regulates its activity (Van Aalten et al., 2000). Similar mechanisms for regulation may also exist at the translational level, through the direct binding of TCA cycle enzymes. The yeast IDH2 protein has been found to directly bind to mitochondrially encoded mRNAs to regulate their translation as a way to directly couple the TCA cycle activity to oxidative phosphorylation (Elzinga et al., 1993).

Acetyl-CoA is a critically important metabolite which provides functional connections between many metabolic pathways, from those of glucose and fatty acid metabolism to the formation of acetylcholine, haeme synthesis, and histone acetylation. It is synthesised through the acetylation of CoA by the PDHC at the end of glycolysis and there is substantive evidence indicating that cerebral pantothenic acid deficiency, resulting in reduced CoA levels, is the culprit in causing various metabolic perturbations. The effects of AD on glycolytic proteins could be investigated as further evidence for metabolic defects. Various studies have found a strong correlation between the activity of the PDHC and acetyl-CoA levels (Szutowicz et al., 1996, 2013). It is unlikely that acetyl-CoA is deficient due to a pyruvate deficiency, as significant probabilities of decrease are seen in proteins which use CoA as a cofactor, whereas the probability of activity of the pyruvate kinase is found to be increased significantly in multiple brain regions in AD cases (Bigl et al., 1999). Thus, the most likely conclusion is that there is reduced CoA in AD brains caused by pantothenic acid deficiency.

In conclusion, in contrast to the prevailing view that AD is caused primarily by Aβ/tau aggregation, we propose that metabolic defects leading to decreased cellular energy metabolism in the brain may be causative of AD-evoked neurodegeneration.

## Data Availability Statement

The datasets presented in this study can be found in online repositories. The names of the repository/repositories and accession number(s) can be found below: http://www.manchester.ac.uk/dementia-proteomes-project, dedicated website.

## Author Contributions

CS, SP, and DH interpreted data and wrote the manuscript. RU performed research, developed the Bayesian data analysis, and read and revised the manuscript. AD developed the Bayesian data analysis, and read and revised the manuscript. JX performed research, analysed data, and revised the manuscript. GC conceived the study idea, wrote the manuscript, and bears overall responsibility for the integrity of this manuscript and of the study. All authors contributed to the article and approved the submitted version.

## Conflict of Interest

The authors declare that the research was conducted in the absence of any commercial or financial relationships that could be construed as a potential conflict of interest.

## Publisher’s Note

All claims expressed in this article are solely those of the authors and do not necessarily represent those of their affiliated organizations, or those of the publisher, the editors and the reviewers. Any product that may be evaluated in this article, or claim that may be made by its manufacturer, is not guaranteed or endorsed by the publisher.

## Abbreviations

sAD: Sporadic Alzheimer’s disease
CoA: Acetyl-coenzyme A
PDHC: Pyruvate dehydrogenase complex
PKAN: Pantothenate kinase-associated neurodegeneration
ChAT: Choline acetyltransferase
OGDHC: 2-oxoglutarate dehydrogenase complex
IDH: Isocitrate dehydrogenase.
