# Supplementary material for: Coenzyme A-Dependent Tricarboxylic Acid Cycle Enzymes Are Decreased in Alzheimer’s Disease Consistent With Cerebral Pantothenate Deficiency
Source: Front Aging Neurosci. 2022 Jun 10;14:893159. doi: 10.3389/fnagi.2022.893159 (PMC9232186; doi:10.3389/fnagi.2022.893159)

Supplementary Methods

These Supplementary Methods were in large part developed and published in our previous manuscripts (see for example, Xu et al., 2019). They are reproduced here for ease of access for the reader.

Protein extraction and preparation for iTRAQ labelling

Protein extraction and preparation for iTRAQ was carried out according to the previously described method ((Xu et al., 2019) and references therein), where each brain region was analysed independently. Brain tissue samples of 100 ± 5 mg were extracted in 5 µL. 1 M triethylammonium bicarbonate buffer (TEAB) + 0.1% (w/v) sodium dodecyl sulphate (SDS) and homogenised at 25 Hz (2 x 3 min; TissueLyser, Qiagen). Tubes were then vortexed for 10 s and centrifuged at 4 °C (5 min, 13,400 x g). Supernatants were transferred into new tubes and protein concentrations determined (Bradford assay; Bio-Rad Protein Assay Dye Reagent Concentrate) with a plate-reader (SpectraMax M5, Molecular Devices). From each sample, a volume equivalent to 100 µg protein was transferred into a new set of tubes for further processing. Identical reference pool samples (total of 100 µg protein per reference sample) were made by combining portions from four representative individual samples from each group, AD, and control. All samples were equalised for final volume using 1 M TEAB + 0.1% (w/v) SDS.

Protein digestion and iTRAQ labelling

Samples were reduced (0.1 volume of 50 mM dithiothreitol) followed by incubation (60 °C, 30 min). Alkylation was performed by addition of 0.05 volumes of 200 mM iodoacetamide, followed by incubation (dark, room temperature, 10-15 min). Protein digestion was performed overnight (37 °C, 10 µg of modified porcine trypsin, Promega) resuspended in 1M TEAB, ensuring the final SDS concentration fell below 0.05% (w/v). After digestion, the samples were dried completely (Eppendorf concentrator), and re-suspended (30 µL 1M TEAB) to achieve equal volumes across all samples before iTRAQ labelling. The iTRAQ labelling was carried out according to the manufacturer's instructions using the 8-plex iTRAQ kit (AB Sciex). Briefly, vials containing iTRAQ reagent were thawed on the bench for 2-3min. After pulse centrifugation, 70 µL isopropanol was added to each vial, followed by a further pulse spin. Contents were then transferred to the protein samples and incubated (r.t., 2-3 h). Each 8-plex contained two separate digests of the reference pool sample, three AD samples, and three control samples. iTRAQ-labelled samples destined for the same liquid chromatography/tandem mass spectrometry (LC-MS/MS) run were pooled, then centrifuge (13,400 x g, 5min). Each pooled sample was then divided into two equal aliquots and dried completely (Eppendorf centrifugal evaporator-concentrator). One pooled aliquot from each 8-plex experiment was subjected to high-pH reverse phase (HpHRP) for peptide fractionation. Remaining dried-pool aliquots were stored at -80 °C for repeated analysis if required.

Supplementary data acquisition by low-pH LC-mass spectrometry

The methodology used here was as previously described and is otherwise as presented here in the Suppl. Methods.
Each fraction was resuspended in 27 µL of 97% water/3% acetonitrile/0.1% trifluoroacetic acid (TFA; v/v/v) and 9 µL was injected into a nano-Acquity UPLC system (Waters). Peptides were

trapped on a nanoACQUITY 2G-V/M Trap Sym C18 5 µm 180 µm x 20 mm (Waters) and washed (flow-rate = 7.5 µL/min, 10 min). Peptides were then eluted and chromatographed using a nanoACQUITY BEH300 C18 1.7 µm 75 µm x 250 mm (Waters) at 300 nL/min using following gradient profile (minutes:%B): 0:3, 3:3, 91:40, 93:90, 108:90, 109:3, 130:3. Buffers used were: buffer A: 97% water + 3% acetonitrile + 0.1% formic acid and buffer B: 100% acetonitrile + 0.1% formic acid (v/v). The eluent was directed into an ESI microionspray II source of a QSTAR Elite Q-TOF spectrometer (AB SCIEX) scanning in MS from 400 to 1200 m/z. Multiply charged peptides (2+ to 4+) were selected for MS/MS analysis (110-1200 m/z). The information-dependent acquisition (IDA) settings were: four precursors per cycle and cycle times (MS 0.75 s, MS/MS1 0.75 s, MS/MS2 0.75s, MS/MS3 1 s and MS/MS4 1 s). Selected peptides were fragmented twice and then dynamically excluded for 90 s. The resulting data were searched against the human component of the Swissprot database (release 2013_03) using Protein-Pilot v4.0 (AB SCIEX). Search parameters were: iTRAQ 8-plex, trypsin; cys alkylation, iodoacetamide; search effort, thorough. A total of 40,466 proteins were searched. To perform FDR analysis on the protein identification, the search database was reversed and concatenated with the forward database and used as the search DB within ProteinPilot. FDR was determined by calculating the number of reverse 'hits' as a proportion of 'forward' hits using the dedicated worksheet exported from the search software.

Supplementary data processing

Methodologies are generally as we previously described (see Xu et al., 2019). Excerpts from this reference have been reiterated here to support the arguments presented in the main manuscript; quotations from the above reference are included in the following text where they are designated by inverted commas.

"Bayesian protein-level differential quantification was performed separately for each brain region using v 1.0.0 of the in-house developed software BayesProt (https://github.com/biospi/bayesprot/release/tag/v1.0.0). Analysis of each brain region in isolation adds strength to the comparison of protein expression changes across multiple regions, as these were identified and quantified independently."

"Since iTRAQ measurements from Time-of-Flight instruments are recorded as discrete ion counts, and technical/biological variation are assumed log-normal, we adopted a generalised linear mixed model (GLMM) with Poisson likelihood and log-link, where each protein was modelled separately using peptide measurements unique to that protein. The sample normalisation factors represent the mass spectrometer's exposure to each sample, and hence were included as a fixed offset within the model. The current version of BayesProt additionally:

(i) Enables estimation of both biological and digestion variance through the incorporation of multiple digests for a single sample (i.e., the six reference pool digests);
(ii) Negates the need for Protein-Pilot normalisation by implementing a two-stage GLMM; and
(iii) Provides a simplified Markov Chain Monte Carlo (MCMC) mixing criterion for both stages."

"In both stages: (a) for each peptide a separate random digest effect is fitted, which has the effect of weighting each peptide's contribution to the protein-level quantification by its reproducibility

across digests; (b) the set of measurement channels within each iTRAQ spectrum are each assigned (i) a baseline fixed effect to account for varying selection/ionisation/fragmentation efficiencies across spectra, and (ii) an independent log-normal residual variance to account for over-dispersion due to background contamination and incorrectly identified spectra. In stage one, we also model the interaction between LC-MS/MS run and iTRAQ channel as a fixed effect, i.e., within each run, we infer the protein-level log ratio between iTRAQ channel 113 and channels, 114, 115, 116, 117, 118, 119 and 121. For each channel relative to 113, the result is a set of posterior probability distributions, one for each protein in the study; these are combined to derive a posterior distribution for the median log ratio for each channel relative to 113, which is taken as the inferred sample normalisation factors."

PCA analysis and pathway analysis

"To construct the PCA plots (see Suppl. Fig. 1 of Xu et al., 2019) the protein-level log ratios for all proteins with measurements across all three 8-plexes were first normalised using the sample normalisation factors. Subsequently, for each protein 'variable', the resulting sample 'observations' were then centred and scaled by the mean standard deviation of their posterior distributions, before final input into the R 'prcomp' function to generate the principal components. In stage two, rather than using point estimates of the normalisation factors as fixed sample offsets, a set of sample fixed effects are fitted, which have prior distributions set to the means and variances of the inferred median log-ratio distributions. In addition, in stage two we specify the full experimental design: (a) protein-level differential expression fold change between cases and controls is fitted as a condition fixed effect (with control as baseline); (b) due to unequal biological variance across cases and controls, subject is treated as two random effects, one for control samples and one for cases. Using the inferred posterior distribution of the condition fixed effect, we performed a one-sided significance test on the posterior probability that the mean fold change is either above or below ± 1.05 – i.e., at least a 5% change from control – denoted as P (1.05 fc). The reciprocal of this posterior probability represents the local FDR (lFDR), the probability that this specific test is a false discovery. In this study, we defined significance using a global FDR threshold of 5%, i.e., the largest set of proteins with an average lFDR ≤ 5% were deemed significant and hence delivered to downstream pathway analysis. The condition fixed effect posterior distributions, FDRs and descriptive statistics (mean log ratio plus 95% highest posterior density interval) for every protein across all regions are presented online (www.manchester.ac.uk/dementia-proteomesproject). Posterior distributions of per-sample protein quantifications are also presented, derived from the latent variables of the sample random effects."

Bayesian analysis

"Residual variances were assigned inverse-Gamma priors, whereas random effects were assigned parameter-expanded Cauchy priors. The model was tested with different prior scale factors to establish that the priors were not informative to the outcome. In stages one and two, the model was run with 10 and 100 MCMC chains per protein, respectively, each chain consisting of 10,000 samples preceded by 3,000 burn-in samples. Mixing was assessed using Warners & Raftery's MCGibbsit run-length diagnostic, combining the estimate error-bounding approach of Raftery and Lewis with the between-chain variance verses within-chain variance approach of Gleman and Rubin (https://cran.r-project.org/web/packages/mcgibbsit/index.html).

For a protein to be considered quantified sufficiently well to be included in downstream pathway, correlation and comparative analyses, we require identification and quantification from at least three spectra. This quality control is important when making comparisons across datasets as it ensures that only high-quality protein quantitation is taken forward into comparative studies, reducing 'noise'."

Data analysis methodologies were otherwise as described in (Xu et al., 2019).

Supplementary Figures

Suppl. Fig. 1
Identification of components of an exemplary Bayesian posterior probability distribution plot.

Suppl. Fig. 2
Expression of cerebral TCA cycle enzymes as presented by Bayesian posterior probability distributions for all brain regions.

Supplementary Tables

Suppl. Table 1.
Clinical characters of AD and control brains used in this study

Suppl. Table 2.
Multiregional Bayesian-differential quantification for cerebral expression of additional pyruvate dehydrogenase proteins.

Suppl. Table 3.
Multiregional Bayesian-differential quantification for cerebral expression of additional isocitrate dehydrogenase proteins.

Suppl. Table 4.
Multiregional Bayesian-differential quantification for cerebral protein expression of additional 2-oxoglutarate dehydrogenase and succinyl-CoA synthetase proteins.

Suppl. Table 5.
Multiregional Bayesian-differential quantification for TCA cycle proteins of aconitase.

Suppl. Table 6.
Multiregional Bayesian-differential quantification for cerebral protein expression of succinate dehydrogenase.

Suppl. Table 7
Multiregional Bayesian-differential quantification for cerebral protein expression of fumarate hydratase and malate dehydrogenase.

Suppl. Table 1.
Clinical characters of AD and control brains used in this study

Case no	Class	Age/sex	Cause of death	Braak stage	Amyloid load	PMD (h)	Brain weight (g)
1	AD	60/M	Alzheimer’s disease	VI	3/3	7	1020
2	AD	62/F	Alzheimer’s disease	VI	3/3	6	831
3	AD	63/F	Bronchopenumonia	VI	2/3	7	1080
4	AD	70/F	Lung cancer	V	3/3	7	1044
5	AD	73/M	Gastrointestinal haemorrhage	IV	3/3	4	1287
6	AD	74/F	Metastatic cancer	V	3/3	8.5	1062
7	AD	74/M	Pseudomonas bacteraemia	VI	2/3	12	1355
8	AD	77/M	Myocardial infarction	VI	3/3	4.5	1180
9	AD	80/M	Bronchopenumonia/pulmonary oedema	V	3/3	5.5	1039
10	Control	61/M	Ischaemic heart disease	-	0	7	1258
11	Control	64/F	Pulmonary embolism	-	0	5.5	1260
12	Control	63/F	Ruptured aorta	-	0	12	1280
13	Control	72/F	Emphysema	-	0	9	1230
14	Control	66/M	Ischaemic heart disease	II	3/3[a]	9	1461
15	Control	76/F	Metastatic carcinoma	-	0	12	1094
16	Control	73/M	Ischaemic heart disease	-	0	13	1315
17	Control	78/M	Ruptured abdominal aortic aneurysm	-	0	7.5	1260
18	Control	78/M	Ruptured myocardial infarction	-	0	12	1416

Brain pathology and amyloid load were determined using the scoring system based on Break and Break staging, where a score of 3 was determined by a qualified neuropathologist and cause of death was determined at post-mortem examination. [a]Despite being phenotypically healthy, patient 15 was found retrospectively to have post-mortem signs consistent with AD and was described as A3, B1, C1 using the ‘ABC’ criteria for AD neuropathology change that incorporates histopathological assessments of Aβ deposits (A), staging of neurofibrillary tangles (B) and scoring and neuritic plaques (C). The corresponding data have been retained in the analysis presented in the article due to the early and asymptomatic nature of this patient.
Abbreviations: AD: Alzheimer’s disease; F: female; M: Male; PMD: *Post-mortem* delay

Suppl. Table 2.
Multiregional Bayesian-differential quantification for cerebral expression of additional pyruvate dehydrogenase proteins.

**PDHA1**<br>**Brain region**	**Peptides**	**Spectra**	**Log2 (fc)**	**Lower**	**Upper**	**Local FDR**
Hippocampus	22	126	-0.264	-0.503	-0.015	0.0525
Entorhinal Cortex	23	140	-0.088	-0.259	0.083	0.4175
Cingulate Gyrus	20	162	-0.126	-0.265	0.017	0.19
Motor Cortex	22	114	-0.088	-0.228	0.053	0.3982
Sensory Cortex	21	148	-0.077	-0.209	0.052	0.456
Cerebellum	19	100	-0.175	-0.292	-0.050	0.0458

**PDHB**<br>**Brain region**	**Peptides**	**Spectra**	**Log2 (fc)**	**Lower**	**Upper**	**Local FDR**
Hippocampus	14	113	-0.342	-0.630	-0.061	0.0287
Entorhinal Cortex	15	88	-0.237	-0.477	0.001	0.0774
Cingulate Gyrus	14	102	-0.202	-0.454	0.040	0.1239
Motor Cortex	8	30	-0.021	-0.129	0.082	0.844
Sensory Cortex	13	129	-0.110	-0.297	0.053	0.316
Cerebellum	12	116	-0.130	-0.298	0.026	0.2098

**PDK2**<br>**Brain region**	**Peptides**	**Spectra**	**Log2 (fc)**	**Lower**	**Upper**	**Local FDR**
Hippocampus	13	113	-0.342	-0.630	-0.061	0.0287
Entorhinal Cortex	15	88	-0.237	-0.477	0.001	0.0774
Cingulate Gyrus	14	102	-0.202	-0.454	0.04	0.1239
Motor Cortex	8	30	-0.021	-0.129	0.082	0.844
Sensory Cortex	13	129	-0.110	-0.297	0.053	0.316
Cerebellum	12	116	-0.130	-0.298	0.026	0.2098

**PDP1**<br>**Brain region**	**Peptides**	**Spectra**	**Log2 (fc)**	**Lower**	**Upper**	**Local FDR**
Hippocampus	2	4	-0.375	-0.819	0.069	0.0717
Entorhinal Cortex	2	4	-0.585	-1.237	0.098	0.0583
Cingulate Gyrus	2	4	-0.234	-0.823	0.259	0.2528
Motor Cortex	1	1	-0.513	-6.227	4.796	0.2856
Sensory Cortex	3	9	-0.354	-0.661	-0.061	0.029
Cerebellum	2	5	-0.414	-0.826	0.0747	0.0646

Suppl. Table 3.
Multiregional Bayesian-differential quantification for cerebral expression of additional isocitrate dehydrogenase proteins.

**IDH1**<br>**Brain region**	**Peptides**	**Spectra**	**Log2 (fc)**	**Lower**	**Upper**	**Local FDR**
Hippocampus	13	40	0.142	-0.055	0.345	0.2185
Entorhinal Cortex	16	41	0.157	-0.070	0.374	0.1971
Cingulate Gyrus	5	20	0.237	0.084	0.386	0.017
Motor Cortex	7	11	0.107	-0.099	0.326	0.3612
Sensory Cortex	9	31	0.108	-0.025	0.247	0.2721
Cerebellum	14	37	0.119	0.001	0.232	0.1858
**IDH3A**<br>**Brain region**	**Peptides**	**Spectra**	**Log2 (fc)**	**Lower**	**Upper**	**Local FDR**
Hippocampus	17	158	-0.380	-0.719	-0.042	0.0359
Entorhinal Cortex	17	106	-0.257	-0.500	-0.009	0.061
Cingulate Gyrus	18	107	-0.293	-0.600	-0.002	0.0623
Motor Cortex	17	70	-0.235	-0.426	-0.032	0.0483
Sensory Cortex	17	160	-0.155	-0.304	-0.003	0.12
Cerebellum	17	110	-0.221	-0.478	0.030	0.1107
**IDH3B**<br>**Brain region**	**Peptides**	**Spectra**	**Log2 (fc)**	**Lower**	**Upper**	**Local FDR**
Hippocampus	12	81	-0.365	-0.701	-0.033	0.0398
Entorhinal Cortex	15	70	-0.206	-0.528	0.109	0.183
Cingulate Gyrus	11	65	-0.230	-0.450	-0.014	0.0634
Motor Cortex	14	51	-0.131	-0.354	0.106	0.2952
Sensory Cortex	13	92	-0.113	-0.274	0.070	0.2993
Cerebellum	12	57	-0.207	-0.505	0.096	0.1674
**IDH3G**<br>**Brain region**	**Peptides**	**Spectra**	**Log2 (fc)**	**Lower**	**Upper**	**Local FDR**
Hippocampus	11	59	-0.471	-0.793	-0.114	0.0118
Entorhinal Cortex	12	52	-0.335	-0.638	0.037	0.0394
Cingulate Gyrus	11	50	-0.322	-0.599	-0.037	0.0359
Motor Cortex	9	30	-0.332	-0.588	-0.092	0.0161
Sensory Cortex	10	54	-0.262	-0.495	-0.029	0.0436
Cerebellum	9	32	-0.309	-0.687	0.058	0.0931

Suppl. Table 4.
Multiregional Bayesian-differential quantification for cerebral protein expression of additional 2-oxoglutarate dehydrogenase and succinyl-CoA synthetase proteins.

OGDHL

Brain region	Peptides	Spectra	Log2 (fc)	Lower	Upper	Local FDR
Hippocampus	10	23	-0.550	-0.833	-0.276	0.0015
Entorhinal Cortex	9	15	-0.472	-0.810	-0.102	0.0132
Cingulate Gyrus	7	9	-0.481	-0.820	-0.141	0.0118
Motor Cortex	6	11	-0.288	-1.130	0.566	0.2684
Sensory Cortex	13	53	-0.559	-0.867	-0.250	0.0038
Cerebellum	10	41	-0.437	-0.879	0.020	0.0498

DLST

Brain region	Peptides	Spectra	Log2 (fc)	Lower	Upper	Local FDR
Hippocampus	14	120	0.104	-0.137	0.349	0.3822
Entorhinal Cortex	12	80	0.256	-0.012	0.534	0.0799
Cingulate Gyrus	13	75	0.073	-0.136	0.283	0.4806
Motor Cortex	13	55	0.020	-0.191	0.228	0.7001
Sensory Cortex	12	99	0.079	-0.037	0.187	0.4333
Cerebellum	16	136	0.101	-0.083	0.281	0.3616

SUCLG1

Brain region	Peptides	Spectra	Log2 (fc)	Lower	Upper	Local FDR
Hippocampus	9	29	-0.817	-1.289	-0.368	0.0021
Entorhinal Cortex	9	33	-0.785	-1.395	-0.242	0.0099
Cingulate Gyrus	8	33	-0.541	-0.860	-0.238	0.0031
Motor Cortex	7	21	-0..626	-1.120	-0.147	0.0137
Sensory Cortex	10	45	-0.605	-0.940	-0.267	0.0029
Cerebellum	10	41	-0.533	-0.923	-0.148	0.0125

SUCLG2

Brain region	Peptides	Spectra	Log2 (fc)	Lower	Upper	Local FDR
Hippocampus	3	8	-0.140	-0.573	0.319	0.3644
Entorhinal Cortex	1	4	-0.361	-1.039	0.232	0.1624
Cingulate Gyrus	1	2	-0.087	-4.450	4.047	0.5339
Motor Cortex	2	3	-0.269	-0.984	0.485	0.2724
Sensory Cortex	3	6	-0.408	-0.702	-0.120	0.0102
Cerebellum	5	9	-0.486	-0.928	-0.038	0.0322

Suppl. Table 5.
Multiregional Bayesian-differential quantification for TCA cycle proteins of aconitase

**ACO1**<br>**Brain region**	**Peptides**	**Spectra**	**Log2 (fc)**	**Lower**	**Upper**	**Local FDR**
Hippocampus	40	181	-0.298	-0.476	-0.091	0.0113
Entorhinal Cortex	46	172	-0.199	-0.445	0.036	0.1266
Cingulate Gyrus	27	120	-0.122	-0.243	0.001	0.1892
Motor Cortex	29	71	-0.134	-0.263	0.001	0.1538
Sensory Cortex	34	171	-0.065	-0.188	0.071	0.5334
Cerebellum	51	198	0.018	-0.117	0.157	0.7918

**ACO2**<br>**Brain region**	**Peptides**	**Spectra**	**Log2 (fc)**	**Lower**	**Upper**	**Local FDR**
Hippocampus	4	9	-0.247	-0.539	0.062	0.1137
Entorhinal Cortex	7	11	-0.393	-0.679	-0.117	0.0134
Cingulate Gyrus	5	7	-0.098	-0.285	0.092	0.3661
Motor Cortex	1	2	0.027	-0.327	0.358	0.6184
Sensory Cortex	7	11	0.038	-0.136	0.202	0.648
Cerebellum	7	11	-0.045	-0.288	0.216	0.5945

Suppl. Table 6.
Multiregional Bayesian-differential quantification for cerebral protein expression of succinate dehydrogenase.

**SDHA**<br>**Brain region**	**Peptides**	**Spectra**	**Log2 (fc)**	**Lower**	**Upper**	**Local FDR**
Hippocampus	16	79	-0.109	-0.421	0.215	0.4021
Entorhinal Cortex	26	107	-0.037	-0.313	0.246	0.6019
Cingulate Gyrus	20	89	0.010	-0.167	0.180	0.7714
Motor Cortex	20	64	-0.031	0.208	0.139	0.6977
Sensory Cortex	21	106	-0.063	-0.159	0.034	0.5593
Cerebellum	20	86	0.003	-0.117	0.131	0.8792

**SDHB**<br>**Brain region**	**Peptides**	**Spectra**	**Log2 (fc)**	**Lower**	**Upper**	**Local FDR**
Hippocampus	14	56	-0.124	-0.454	0.200	0.3571
Entorhinal Cortex	17	58	-0.030	-0.346	0.282	0.6104
Cingulate Gyrus	12	52	-0.028	-0.166	0.116	0.7429
Motor Cortex	9	33	-0.048	-0.204	0.121	0.6209
Sensory Cortex	13	48	-0.056	-0.187	0.059	0.6169
Cerebellum	14	60	0.029	-0.126	0.186	0.7209

**SDHD**<br>**Brain region**	**Peptides**	**Spectra**	**Log2 (fc)**	**Lower**	**Upper**	**Local FDR**
Hippocampus	1	2	0.280	-0.314	0.845	0.1988
Sensory Cortex	1	1	0.062	-5.997	6.386	0.5189
Cerebellum	1	1	-3.586	-31.88	18.67	0.3458

Suppl. Table 7
Multiregional Bayesian-differential quantification for cerebral protein expression of fumarate hydratase and malate dehydrogenase.

**FH**<br>**Brain region**	**Peptides**	**Spectra**	**Log2 (fc)**	**Lower**	**Upper**	**Local FDR**
Hippocampus	24	146	-0.098	-0.331	0.133	0.3942
Entorhinal Cortex	24	127	0.095	-0.076	0.269	0.3727
Cingulate Gyrus	21	93	0.030	-0.107	0.170	0.7423
Motor Cortex	22	67	0.024	-0.151	0.209	0.7068
Sensory Cortex	28	157	0.032	-0.071	0.134	0.7885
Cerebellum	27	109	0.008	-0.084	0.095	0.9216

**MDH1**<br>**Brain region**	**Peptides**	**Spectra**	**Log2 (fc)**	**Lower**	**Upper**	**Local FDR**
Hippocampus	33	335	-0.011	-0.188	0.155	0.7762
Entorhinal Cortex	35	291	0.087	-0.118	0.301	0.4287
Cingulate Gyrus	26	323	0.094	-0.077	0.264	0.3772
Motor Cortex	23	183	0.061	-0.107	0.225	0.5488
Sensory Cortex	32	366	0.102	-0.031	0.235	0.306
Cerebellum	32	259	0.143	0.009	0.276	0.1293

**MDH2**<br>**Brain region**	**Peptides**	**Spectra**	**Log2 (fc)**	**Lower**	**Upper**	**Local FDR**
Hippocampus	26	420	-0.060	-0.500	0.359	0.5198
Entorhinal Cortex	30	345	0.107	-0.160	0.407	0.3869
Cingulate Gyrus	29	464	0.006	-0.201	0.229	0.7482
Motor Cortex	25	279	0.015	-0.159	0.188	0.7601
Sensory Cortex	25	400	0.026	-0.099	0.145	0.782
Cerebellum	26	287	0.094	-0.032	0.211	0.3410

Suppl. Fig. 1
Identification of components of an exemplary Bayesian posterior probability distribution plot.

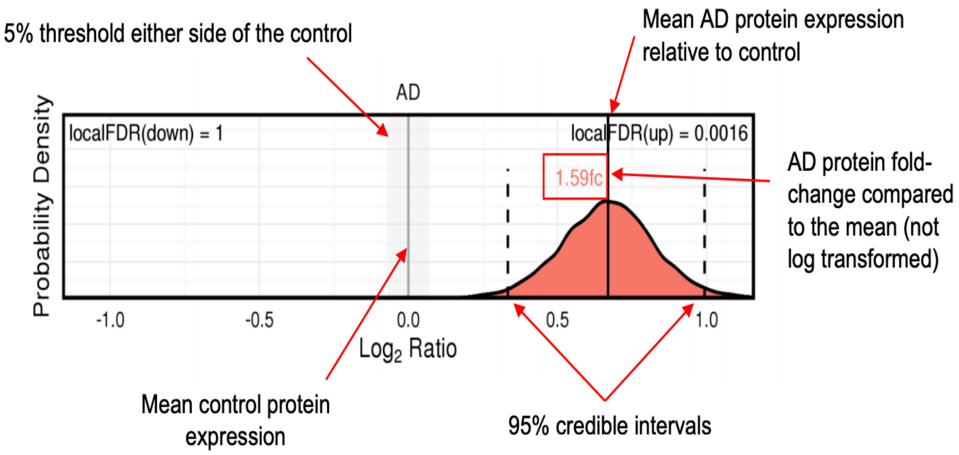

Suppl. Fig. 2

Expression of cerebral TCA cycle enzymes as presented by Bayesian posterior probability distributions for all brain regions. The unique Swiss-Prot IDs for each protein are labelled in brackets. Each plot shows the posterior distribution along with the mean expression ratio and the calculated false-discovery rate (FDR) for each molecule having an up or down-regulation between cases and control of at least 5%.

A Pyruvate Dehydrogenase E1 Subunit Alpha 1 (PDHA1)

B Pyruvate Dehydrogenase E1 Subunit B (PDHB)

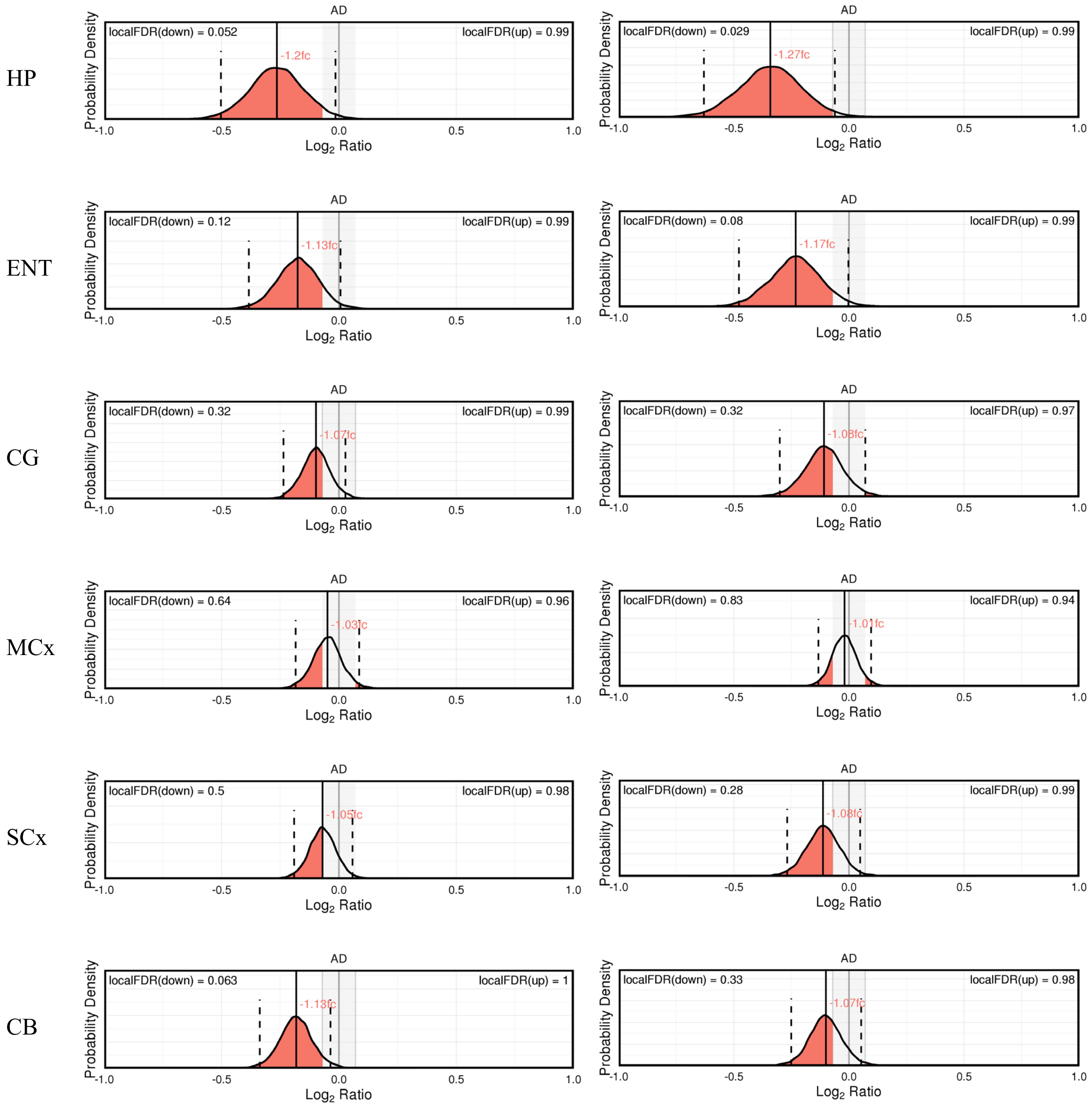

C Pyruvate dehydrogenase kinase isozyme 2 (PDK2)

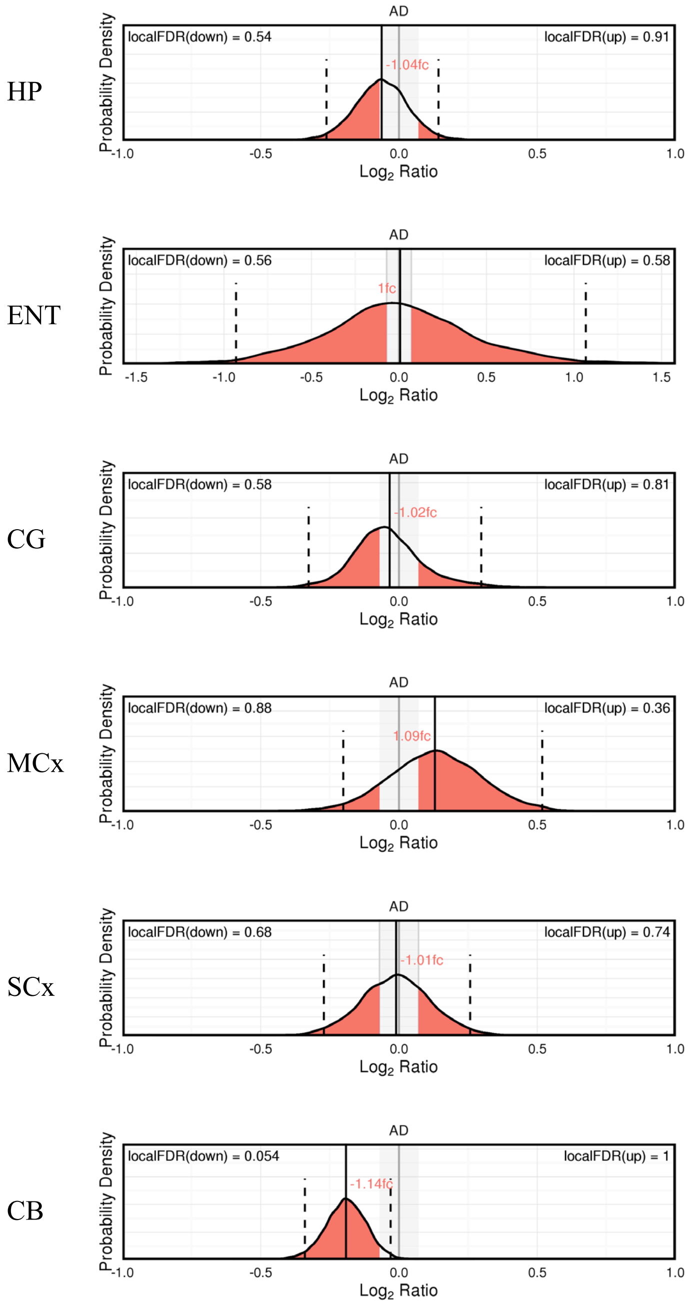

D Pyruvate dehydrogenase-phosphatase 1 (PDP1)

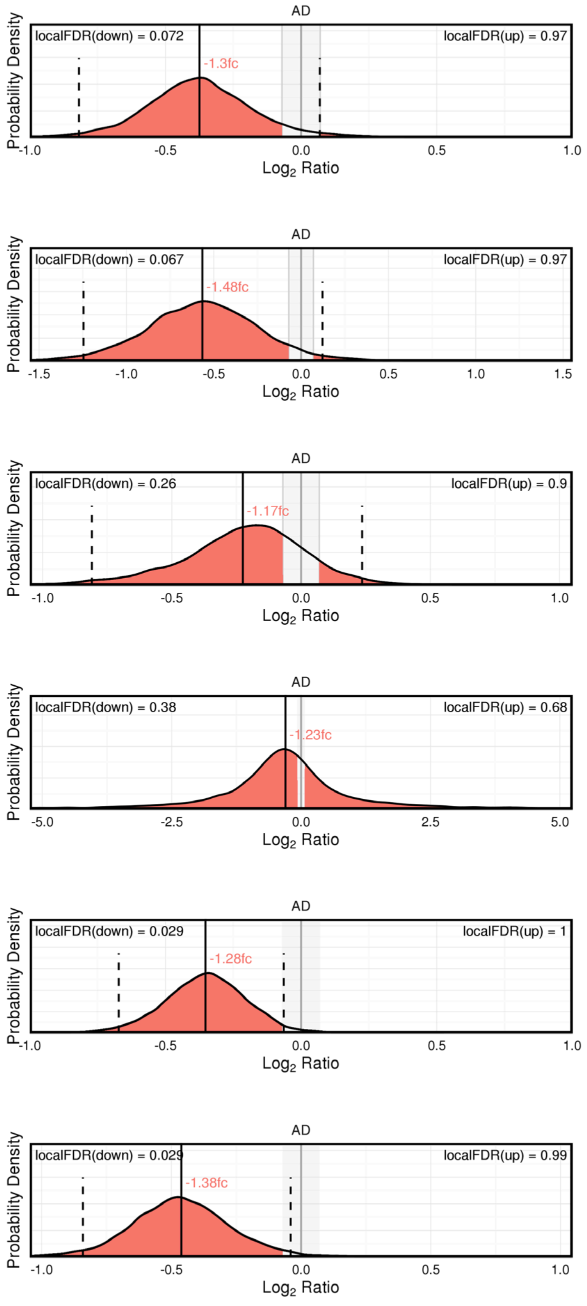

E Isocitrate dehydrogenase [NADP], cytoplasmic (IDH1)

F Isocitrate dehydrogenase [NAD] subunit α, mitochondrial (IDH3A)

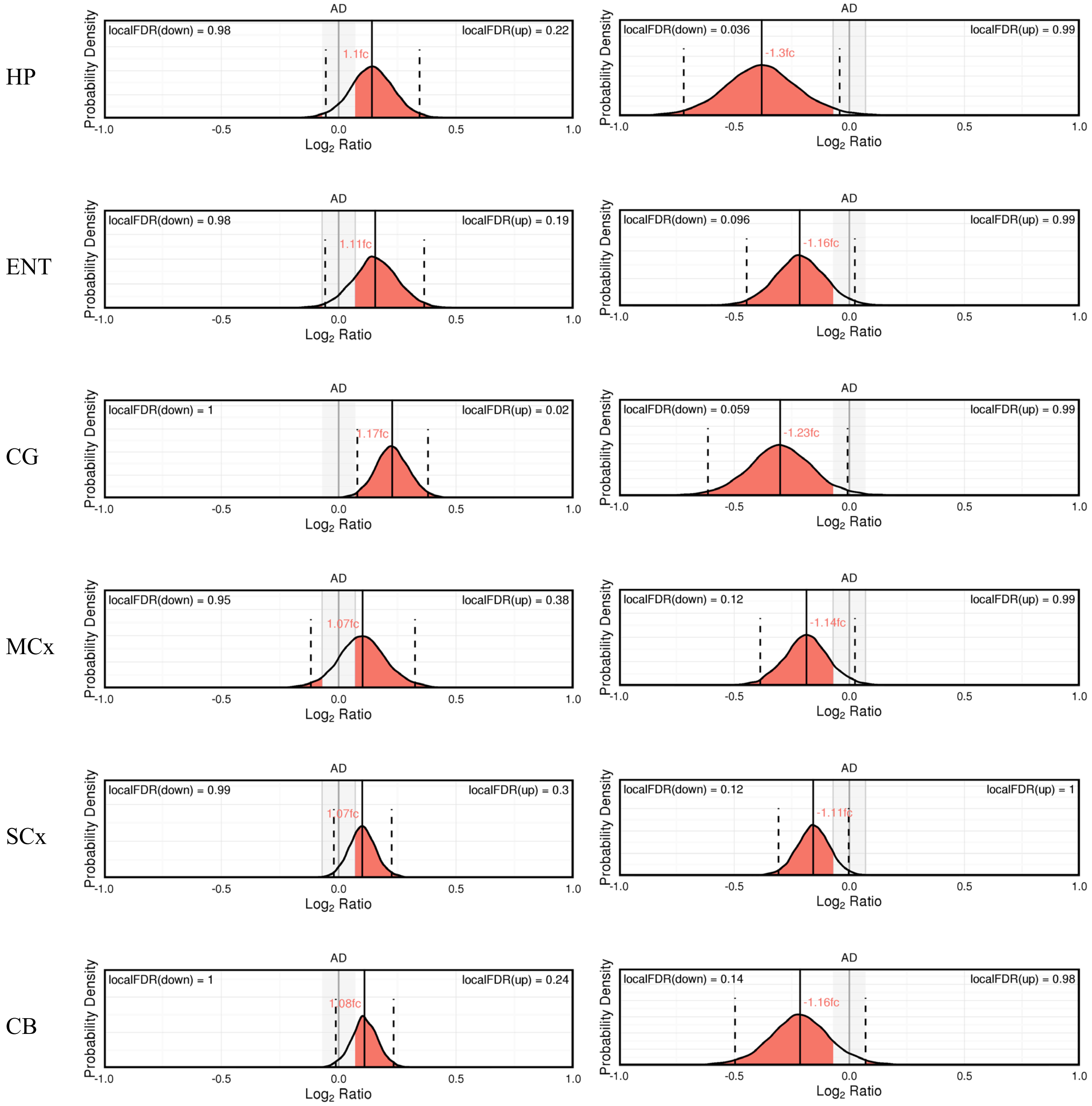

G Isocitrate dehydrogenase [NAD] subunit β, mitochondrial (IDH3B)

H Isocitrate dehydrogenase [NAD] subunit γ, mitochondrial (IDH3G)

HP

ENT

CG

MCx

SCx

CB

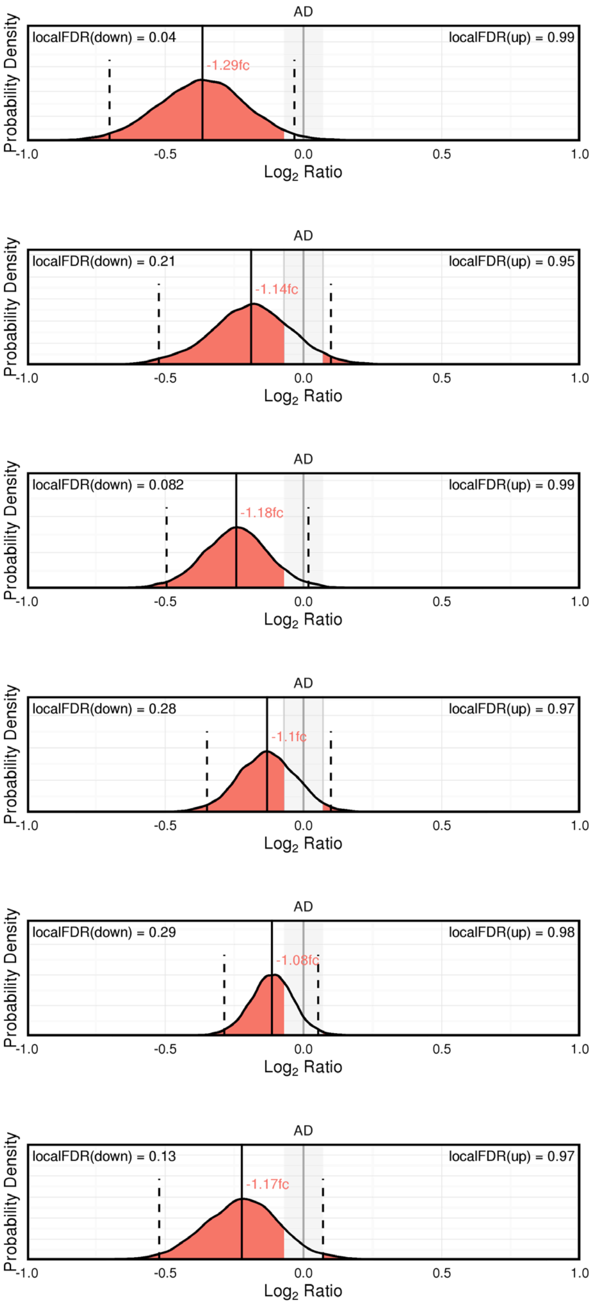

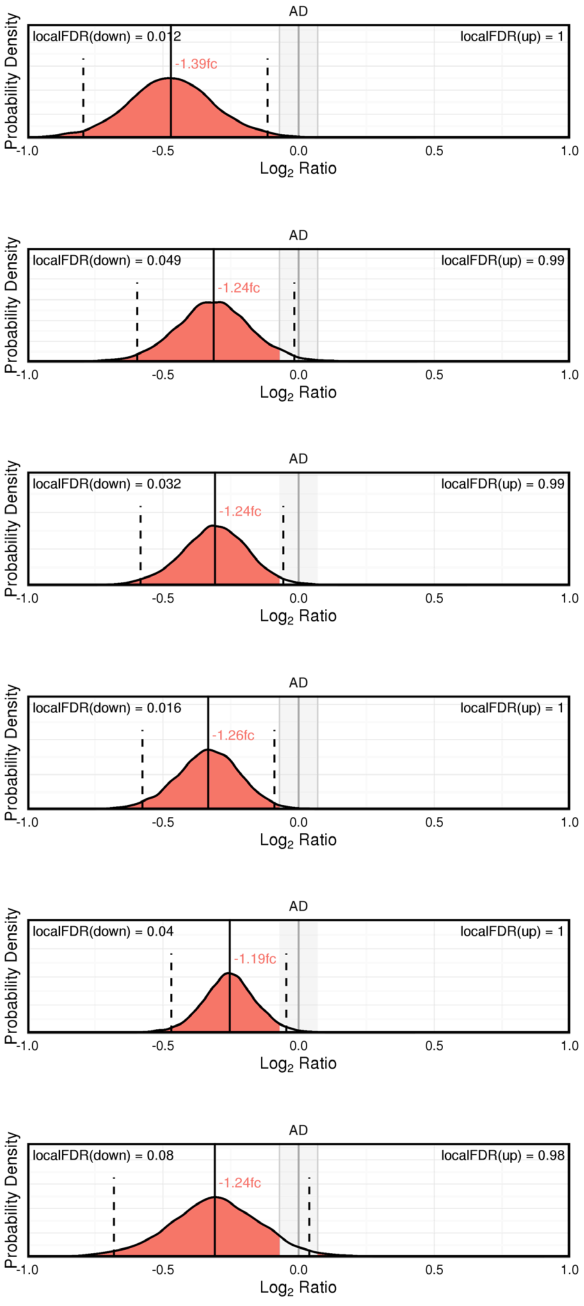

I Succinyl-CoA ligase [GDP-forming] subunit α (SUCLG1)

J Succinyl-CoA ligase [GDP-forming] subunit β (SUCLG2)

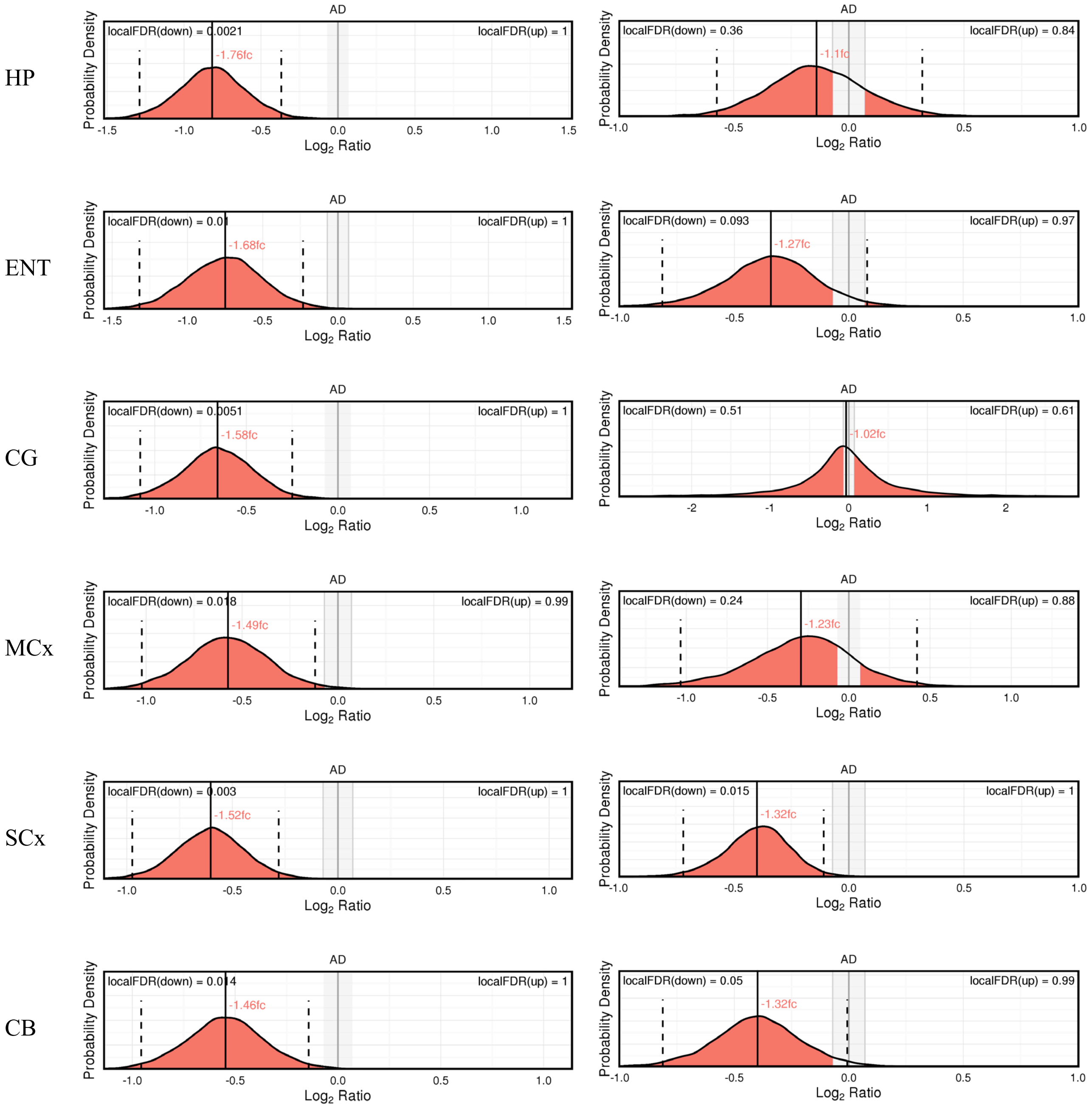

K 2-oxoglutarate dehydrogenase like protein (OGDHL)

L Dihydrolipoyllysine-residue succinyltransferase of 2-oxoglutarate dehydrogenase (DLST)

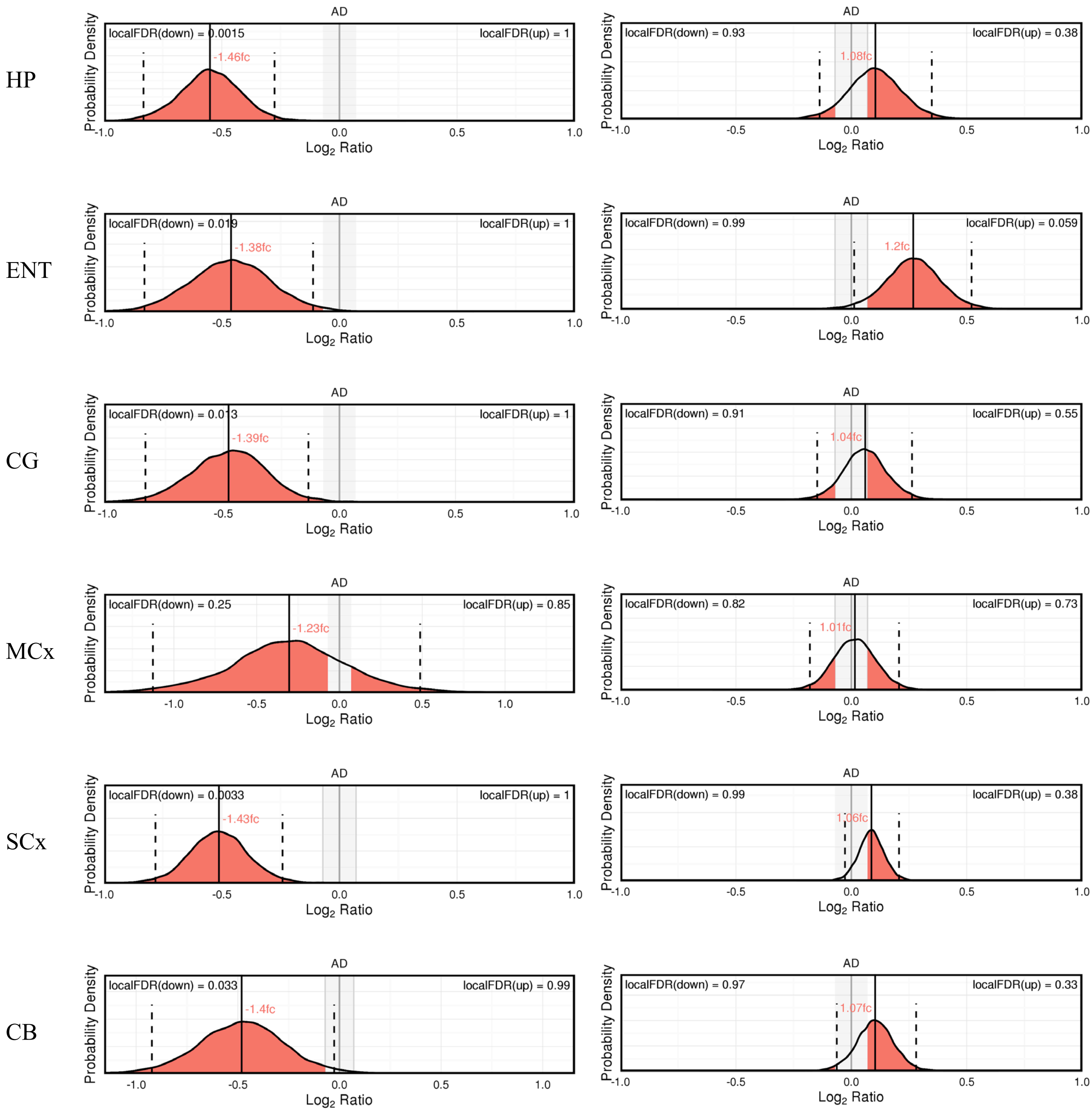

M Aconitate hydratase, cytoplasmic (ACO1) **N** Aconitate hydratase, mitochondrial (ACO2)

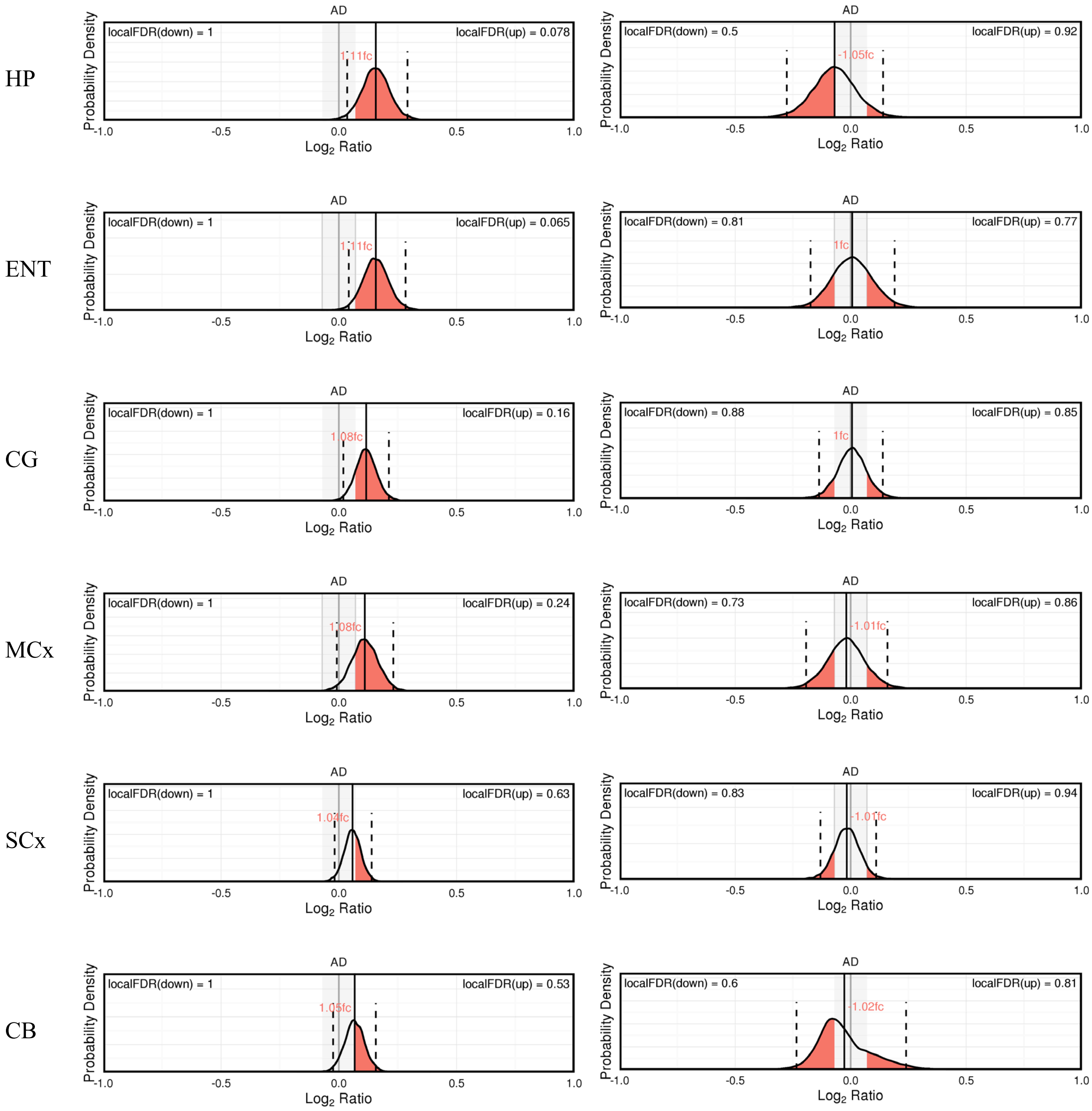

O Succinate dehydrogenase [ubiquinone] flavoprotein subunit (SDHA)

P Succinate dehydrogenase [ubiquinone] iron-sulfur subunit (SDHB)

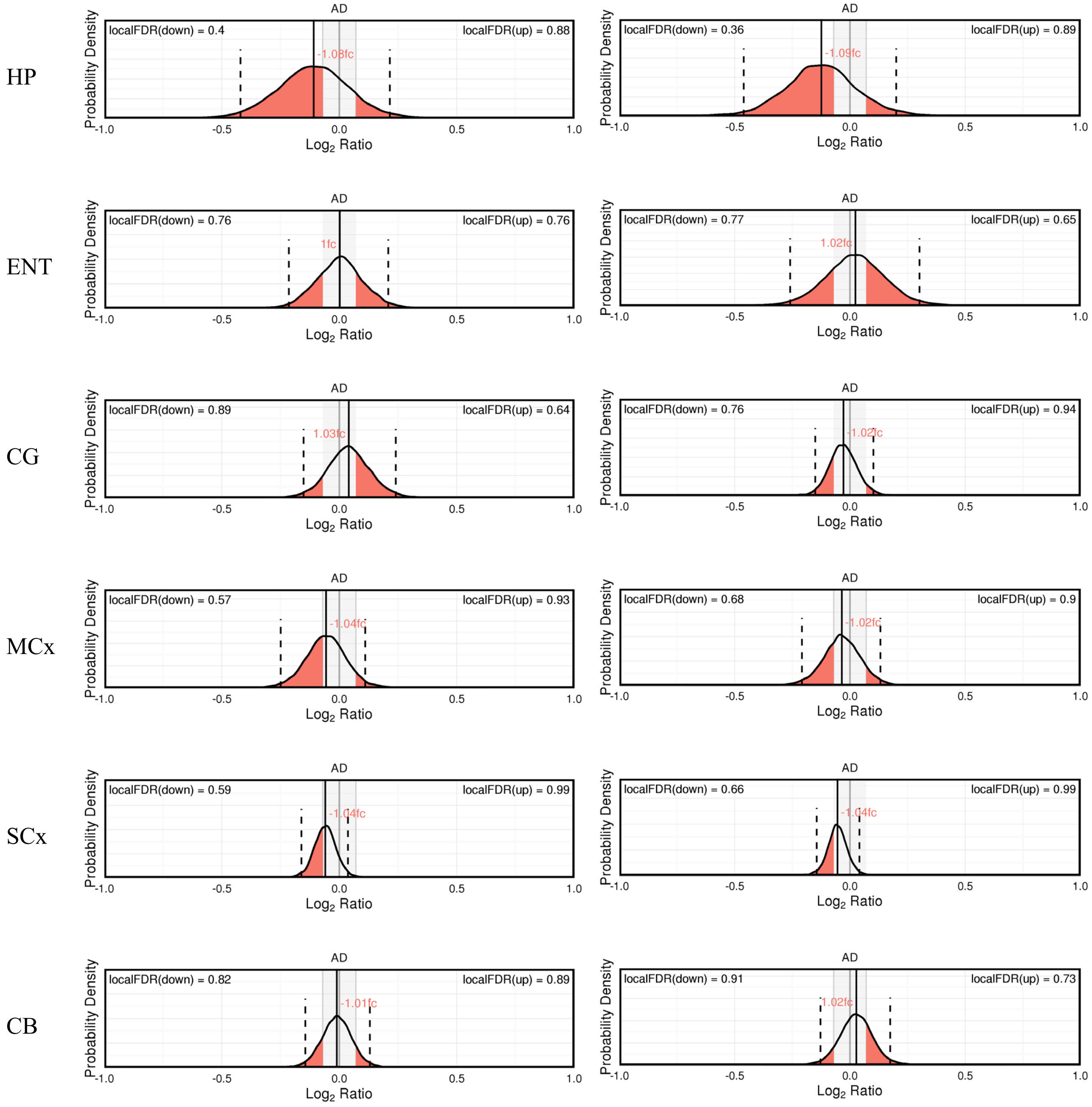

Q Succinate dehydrogenase [ubiquinone] cytochrome b small subunit (SDHD)

R Fumarate hydratase (FH)

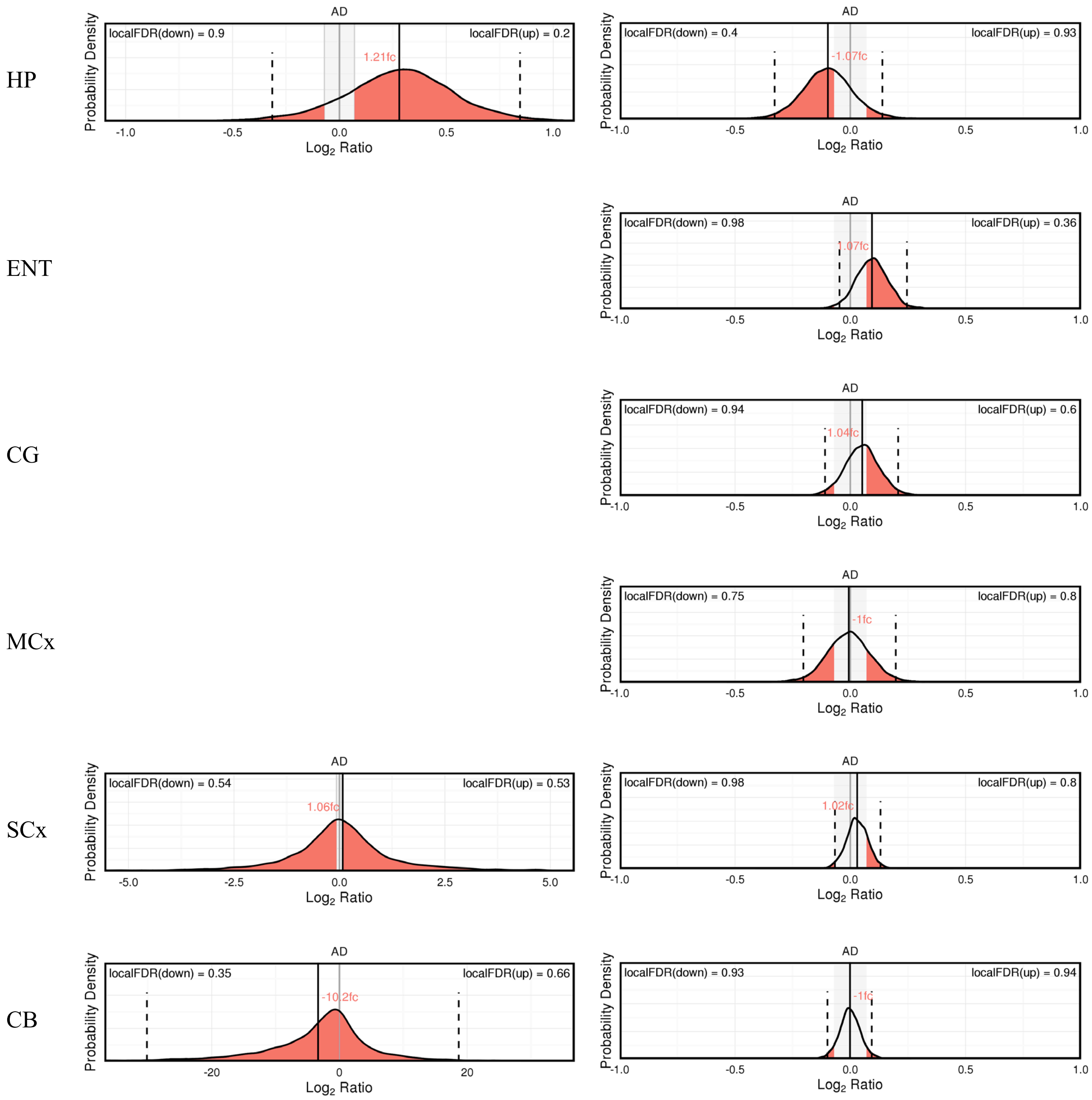

S Cytoplasmic malate dehydrogenase (MDH1) **T** Mitochondrial malate dehydrogenase (MDH2)

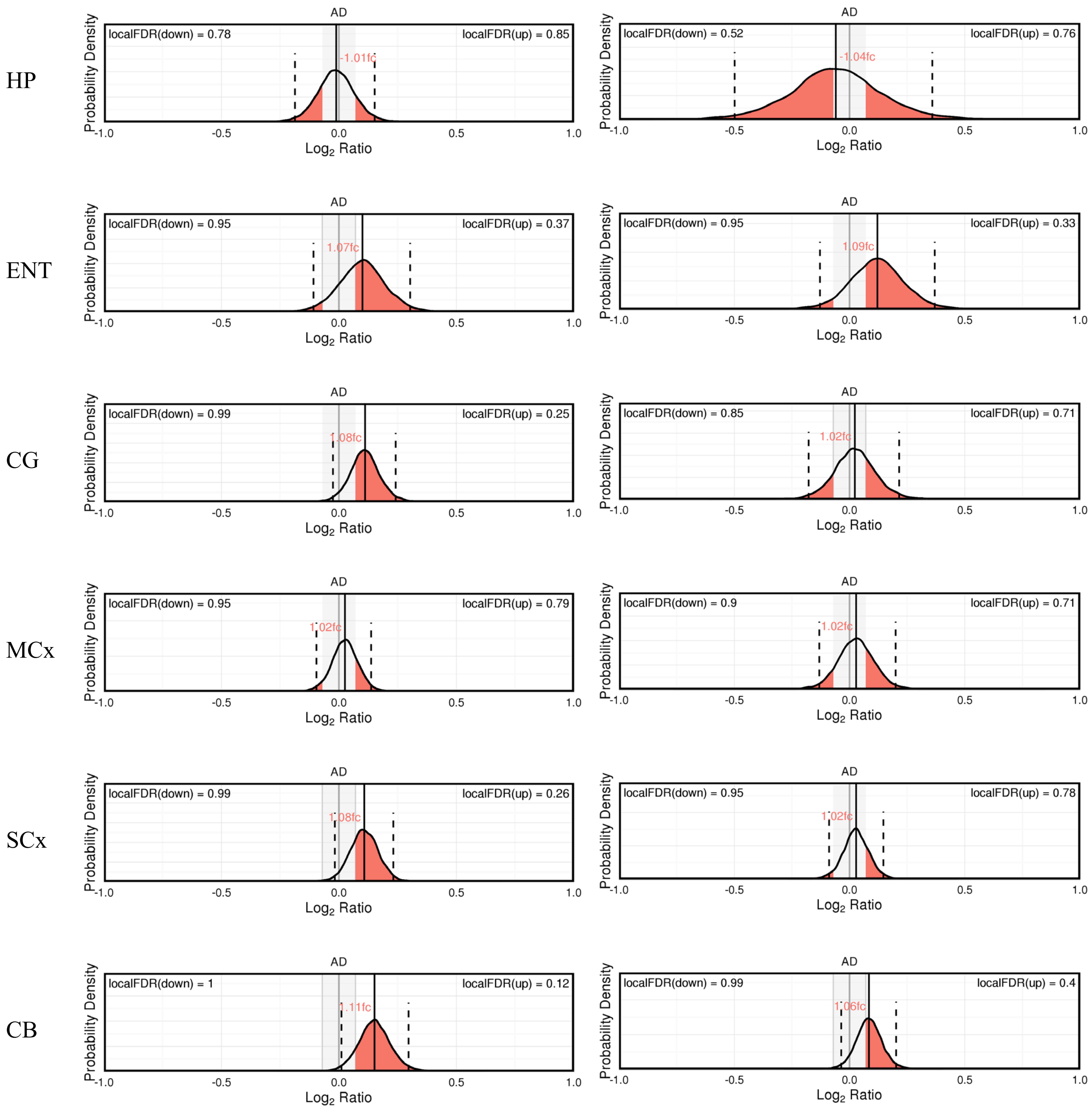

Supplement: Supplementary file 1 [file Data_Sheet_1.PDF]
